# A mathematical model analyzing temperature threshold dependence in cold sensitive neurons

**DOI:** 10.1371/journal.pone.0237347

**Published:** 2020-08-12

**Authors:** Kees McGahan, James Keener

**Affiliations:** Department of Mathematics, University of Utah, Salt Lake City, Utah, United States of America; University of Southern California, UNITED STATES

## Abstract

Here we examine a class of neurons that have been recently explored, the somatosensory neuronal subclass of cold thermosensors. We create a mathematical model of a cold sensing neuron that has been formulated to understand the variety of ionic channels involved. In particular this model showcases the role of TRPM8 and voltage gated potassium channels in setting the temperature dependent activation and inactivation threshold level. Bifurcation analysis of the model demonstrates that a Hodgkin-Huxley type model with additional TRPM8 channels is sufficient to replicate observable experimental features of when different threshold level cold thermosensors turn on. Additionally, our analysis gives insight into what is happening at the temperature levels at which these neurons shut off and the role sodium and leak currents may have in this. This type of model construction and analysis provides a framework moving forward that will help tackle less well understood neuronal classes and their important ionic channels.

## Introduction

With the progress being made in genomics and molecular genetics recently there has never been a better time to explore the nervous system and its disorders. One approach to investigating the nervous system is to focus on individual neurons and determine their contribution to the entire complex. The key to this approach is creating a functional profile of the neurons and breaking them into categories or classes. This technique revolves around either identifying the types of compounds that activate the neuron, or the types of receptors and ionic channels on the cell’s surface [1].

One such classification technique is constellation pharmacology, a technique which was recently utilized to further the understanding of cold thermosensors [2]. These dorsal root and trigeminal ganglion neurons are characterized by responsiveness to ATP, menthol, mustard oil, and a varying range of cold temperatures. This varying range of temperature activation has lead to describing the receptiveness of these neurons to temperature as either high or low threshold. The low threshold cold responsive neurons are typically described to activate between 20°C and 30°C while the high threshold neurons respond to temperatures between 8°C and 20°C [2–5]. These threshold levels have been used to divide cold responsive neurons into two classes: cool thermoreceptors and cold nociceptors [3, 6].

The process of discovering the ion channels that categorize these cold thermosensors has also begun to shed light on this temperature threshold difference. One critical defining feature of these cold sensors was found to be the presence of TRPM8, a channel from the transient receptor potential superfamily [5, 7]. These channels are voltage gated with their open probability influenced by multiple things, but primarily temperature. While TRPM8 has been marked as a critical channel in defining cold responsive neurons, TRPA1 and *Na*_*v*_1.8, a cold insensitive sodium channel, are believed to play roles at extreme low temperatures [5, 6]. In addition to these cold specific ion channels, voltage gated potassium channels have also been hypothesized to influence the temperature threshold level of activation [2, 8]. In particular, it is thought that the ratio of TRPM8 channel to *K*_*v*_1 potassium channel density controls the threshold level. It has been shown that blocking TRPM8 channels can force a neuron to switch to a higher threshold and then applying an additional blocker of *K*_*v*_1 channels can restore the neuron to its initial threshold level [8].

Although a multitude of labs have experimentally examined cold sensing neurons, few attempts have been made to model their behavior mathematically. The first main attempts were of the Huber-Braun cold receptor variety which showed the desired response to temperature, but did not include TRPM8 channel activity or the ability to track sodium and potassium ion fluxes [9]. With the discovery of the importance of TRPM8 channels, models were created and parameters experimentally fit for TRPM8 channels and their activation curves [7]. These models include a version without menthol and two versions with menthol; the Hodgkin-Huxley version and the Monod-Wyman-Changeux type model. The development of a mathematical model of the TRPM8 channel allowed for including this new channel into a modified version of the Huber-Braun model [10]. Although successfully highlighting the importance of TRPM8 in creating a cold thermosensor, this model focuses only on cold sensitive neurons activating above 20°C and leaves out exploring what sets the temperature threshold level for activation and the corresponding role of potassium channels [10].

Here we develop a biologically driven model of a general cold thermosensor that includes the presence of TRPM8 channels. The foundation of this model is the Hodgkin-Huxley model from 1952 with the secondary component being the addition of the TRPM8 model without menthol from the Voets lab [11, 12]. The goals of our model are as follows:

1. to explicitly track differences in temperature dependent response in cold sensitive neurons as it relates to having different ion channel densities
2. to examine how specifically tuned high and low threshold cold sensitive neurons can be with regards to their activating and inactivating temperatures
3. to showcase the ability of simple physiological models to examine biological features and questions in neuroscience that have proven difficult to examine experimentally.

## Materials and methods

The model is composed of a general Hodgkin-Huxley neuron with an additional current through the TRPM8 channel [12]. All combined, this gives the following for membrane voltage *V* with gating variables *m*, *n*, *h*:

$$\begin{array}{c} \frac{dV}{dt}=\frac{1}{Cm}\left(-I_{Na}-I_{K}-I_{l}-I_{m8}\right) \end{array}$$ (1)

$$\begin{array}{c} \frac{dj}{dt}=\Phi \left(T\right)\left[a_{j}\left(V\right)\left(1-j\right)-b_{j}\left(V\right)j\right];j=m,n,h \end{array}$$ (2)

The Hodgkin-Huxley current equations have the form:

$$\begin{array}{c} I_{Na}=g_{Na}m^{3}h\left(V-E_{Na}\right) \end{array}$$ (3)

$$\begin{array}{c} I_{K}=g_{K}n^{4}\left(V-E_{K}\right) \end{array}$$ (4)

$$\begin{array}{c} I_{l}=g_{l}\left(V-E_{l}\right) \end{array}$$ (5)

where *I*_*K*_ is the potassium current, *I*_*Na*_ the sodium current and *I*_*l*_ the leak current. The additional parameters include the equilibrium potentials of the various ions *E*_*Na*_, *E*_*K*_, *E*_*l*_, the maximum ionic conductances *g*_*Na*_, *g*_*K*_, *g*_*l*_ and the membrane capacitance *C*_*m*_. Finally, the rate constants *a*_*j*_ and *b*_*j*_ were experimentally fitted to exponential functions to give the 6 equations below:

$$\begin{array}{c} a_{m}\left(V\right)=\frac{0.1(V_{r}-V+25)}{\text{exp}(\frac{V_{r}-V+25}{10})-1} \end{array}$$ (6)

$$\begin{array}{c} B_{m}\left(V\right)=4\text{exp}\left(\frac{V_{r}-V}{18}\right) \end{array}$$ (7)

$$\begin{array}{c} a_{h}\left(V\right)=0.07\text{exp}\left(\frac{V_{r}-V}{20}\right) \end{array}$$ (8)

$$\begin{array}{c} B_{h}\left(V\right)=\frac{1}{\text{exp}(\frac{V_{r}-V+30}{10})+1} \end{array}$$ (9)

$$\begin{array}{c} a_{n}\left(V\right)=\frac{0.01(V_{r}-V+10)}{\text{exp}(\frac{V_{r}-V+10}{10})-1} \end{array}$$ (10)

$$\begin{array}{c} B_{n}\left(V\right)=0.125\text{exp}\left(\frac{V_{r}-V}{80}\right). \end{array}$$ (11)

The final portion of the Hodgkin-Huxley parameters and equations are *V*_*r*_ which represents the equilibrium resting potential and Φ(*T*) which is a temperature dependent coefficient that adjusts the rate constants from having been experimentally calculated at 6.3°C. The values of all Hodgkin-Huxley parameters are *V*_*r*_ = −65mV, *E*_*Na*_ = *V*_*r*_ + 115mV, *E*_*K*_ = *V*_*r*_ − 12mV, *E*_*l*_ = *V*_*r*_ + 10.613mV, $\Phi (T)=3^{\frac{T-6.3}{10}}$, *g*_*Na*_ = 120mS/cm^2^, *g*_*K*_ = 36mS/cm^2^, *g*_*l*_ = 0.3mS/cm^2^, *C*_*m*_ = 1*μ*F/cm^2^. In addition to the Hodgkin-Huxley base model, we include a current for the cold sensing TRPM8 channel. We give the current the basic form:

$$\begin{array}{c} I_{m8}=g_{m8}a_{m8}\left(V-E_{m8}\right) \end{array}$$ (12)

where, as before, *g*_*m*8_ is the maximal conductance of TRPM8 and *E*_*m*8_ the reversal potential for TRPM8 channels. From the Voets lab we have the open probability of the channel, *a*_*m*8_, is temperature dependent and given by

$$\begin{array}{c} a_{m8}=\frac{1}{1+\text{exp}(\frac{\Delta H-(T+273.15)\Delta S-zFV/1000}{R(T+273.15)})}, \end{array}$$ (13)

where Δ*H* and Δ*S* are the difference in enthalpy and entropy between the open and closed states, *z* is the gating charge, *F* and *R* are Faraday’s and the universal gas constant, and *T* is temperature in degrees Celsius [7, 11]. With *R* and *F* being assigned their classical value, the remainder of the TRPM8 channel parameters were experimentally fit to yield $\Delta H=-156\frac{\text{kJ}}{\text{mol}}$, $\Delta S=-550\frac{\text{J}}{\text{molK}}$, *z* = 0.87, $R=8.3144\frac{\text{J}}{\text{molK}}$, $F=96485\frac{\text{C}}{\text{mol}}$. Note that the parameters to vary are the maximal conductance of the various ion channels, *g*_*m*8_, *g*_*K*_, *g*_*l*_ and *g*_*Na*_ and temperature *T*. These allow us to examine the neuron’s response to temperature and how the relative densities of the ion channels present effect at which temperature level the neurons activate and inactivate. The reversal potential of the TRPM8 channels, *E*_*m*8_ has been experimentally shown to be near 0mV and hence for all analysis we set *E*_*m*8_ = 0 [13].

All bifurcation analysis is done using the software XPPAUT [14]. The temperature ramp and fixed temperature simulations are run in MATLAB [15].

## Results

**Fig 1:**
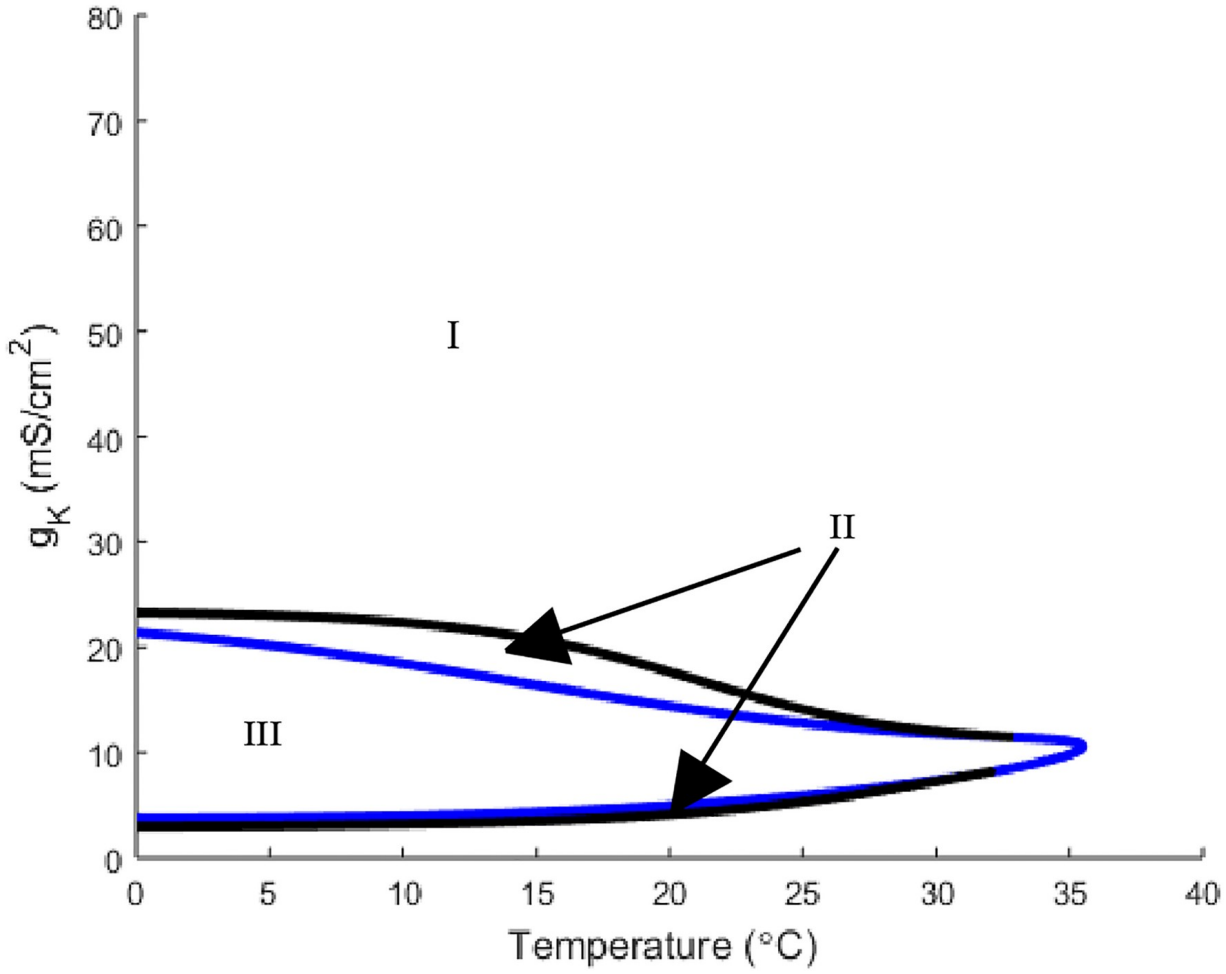
A two parameter bifurcation diagram of the Hodgkin-Huxley model depicting the relationship between temperature and *g*_*K*_. The blue curve represents the Hopf bifurcation while the black curve is a saddle-node-periodic (SNP) bifurcation. In region I there are no oscillatory solutions and only a stable steady state solution. In region II there is a stable and an unstable oscillatory solution in addition to a stable steady state solution. Region III has a stable oscillatory solution coexisting with an unstable steady state solution.

Before analyzing the full model, it is important to see how temperature affects the basic Hodgkin-Huxley model (*g*_*m*8_ = 0). We begin by looking at the temperature and maximal potassium conductance *g*_*K*_ two parameter bifurcation diagram (Fig 1). Under the Hodgkin-Huxley model structure there are two types of stable, and hence observable, solutions. There is a stable steady state solution which occurs at the resting membrane potential and a stable periodic solution which is periodic action potential spiking. Note, the bifurcation diagrams presented in this analysis show separations of parameter space into regions with different solutions types.

This parameter space is broken up into 3 regions by two curves. Region I corresponds to the parameter values that have no oscillatory solutions with only a stable steady state solution for the voltage variable *V*. In region II, sandwiched between the blue and black curves, we have the parameter values for which there exists both stable and unstable oscillatory solutions in addition to a stable steady state solution. In this region, the neuron’s behavior (oscillatory or steady state) is dependent upon the given initial data. Finally, region III, the area bordered by the blue curve, has stable oscillatory solution coexisting with an unstable steady state solution. The curves are indicating the bifurcations that are occurring in parameter space. The black curve is representative of a saddle-node-periodic bifurcation (SNP) that corresponds to the creation of the two (one stable and one unstable) periodic solutions. Additionally, the blue curve is the Hopf bifurcation that corresponds to the transition to a stable oscillatory solution and unstable steady state. The final regions of interest are the meeting points between the SNP (black) and Hopf (blue) curves which are codimension 2 bifurcation points.

**Fig 2:**
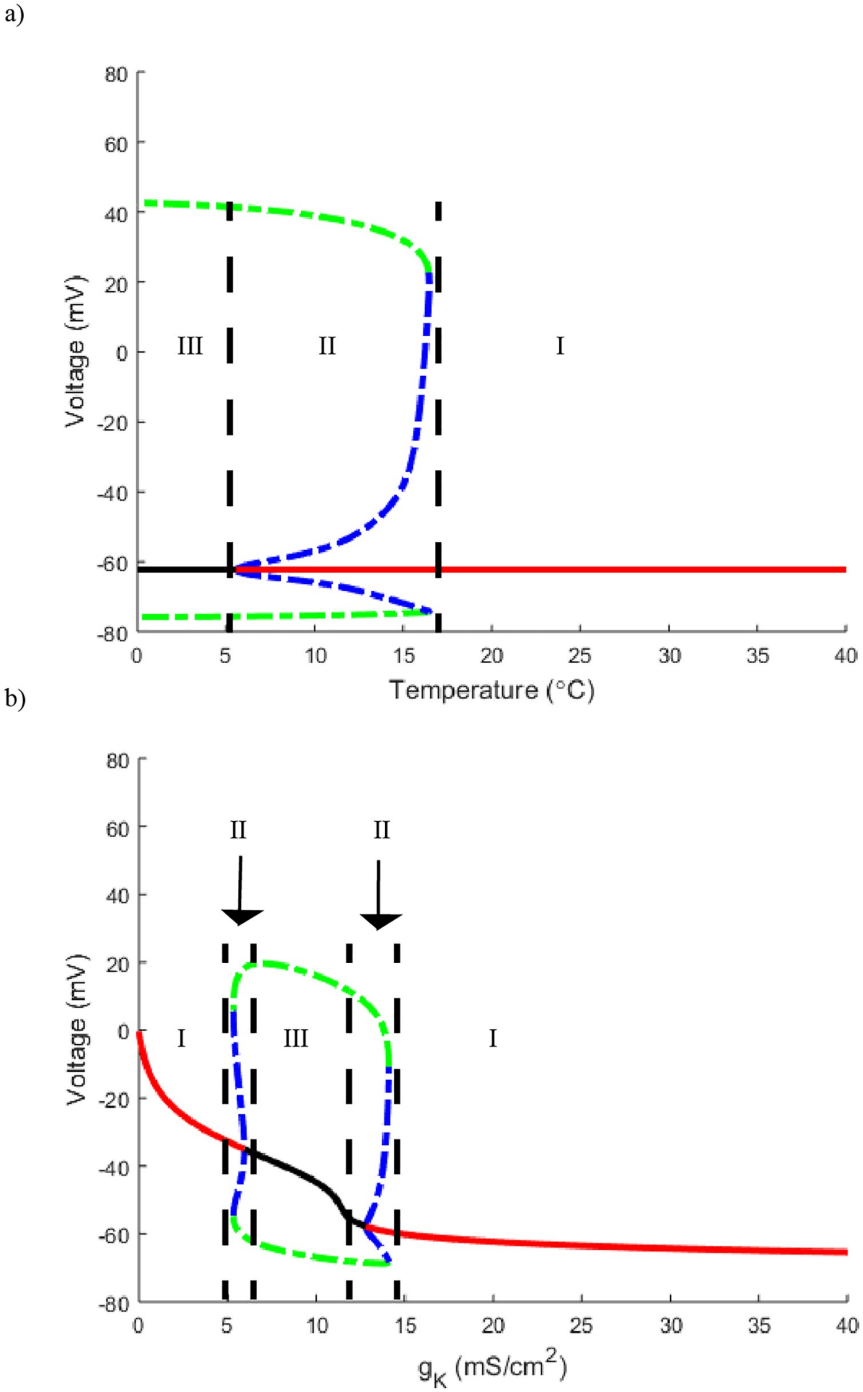
One parameter bifurcation slices of Fig 1. a) Horizontal slice through Fig 1 with *g*_*K*_ set to 20, b) a vertical slice with temperature set to 25°C. The red curve corresponds to a stable steady state while the solid black curve represents an unstable steady state. The meeting point corresponds to a Hopf bifurcation with the blue and green curves denoting the amplitudes of the unstable and stable oscillatory solutions respectively. Regions are divided by dashed black lines. Region I corresponds to a regime with a single, stable steady state solution. In region II we have a stable steady state solution with a stable (green curve) and unstable (blue curve) oscillatory solution. In region III the oscillatory solution is stable with an unstable steady state solution.

Analysis can be done on these bifurcation plots by fixing one parameter at specific values and then observing the dynamics of *V* as the other parameter changes. Starting at the standard Hodgkin-Huxley parameter values namely, *g*_*K*_ = 36, the neuron has a stable steady state for all temperature values which is seen by noting that the neuron is in region I. Consider a vertical slice with temperature set to 15°C and *g*_*K*_ = 36. As *g*_*K*_ is decreased, the neuron crosses the black (SNP) curve in Fig 1 from region I to II, corresponding to the appearance of two oscillatory solutions, one unstable and one stable. Then continuing to decrease *g*_*K*_ leads the neuron to cross the blue (Hopf) curve into region III resulting in the unstable oscillatory solution disappearing. This analysis is encapsulated by the horizontal and vertical one parameter slices of this bifurcation diagram depicted in Fig 2.

The one parameter slices contain three regions of interest and four curves to keep track of. The regions are determined by the varying parameter along the *x* axis with all other parameters fixed for these plots. In region I we have only a stable steady state solution indicated by the red curve. In region II the steady state is still stable but there is an appearance of a stable oscillatory solution whose amplitude is indicated by the green curve and an unstable oscillatory solution which has an amplitude indicated by the dotted blue curve. The final region III transitions to an unstable steady state with a stable periodic solution whose amplitude is the green dashed curve. These slices also showcase the Hopf bifurcation point which occurs at the meeting between black and red curves and a SNP point which occurs at the meeting between blue and green curves. Fig 2 shows more explicitly the features of the neuron’s membrane potential, *V*, in response to parameter changes. It is important to note that for the top panel in Fig 2, *g*_*K*_ was decreased to 20 for any activation of the neuron to occur while recalling that the standard Hodgkin-Huxley value of *g*_*K*_ is 36.

**Fig 3:**
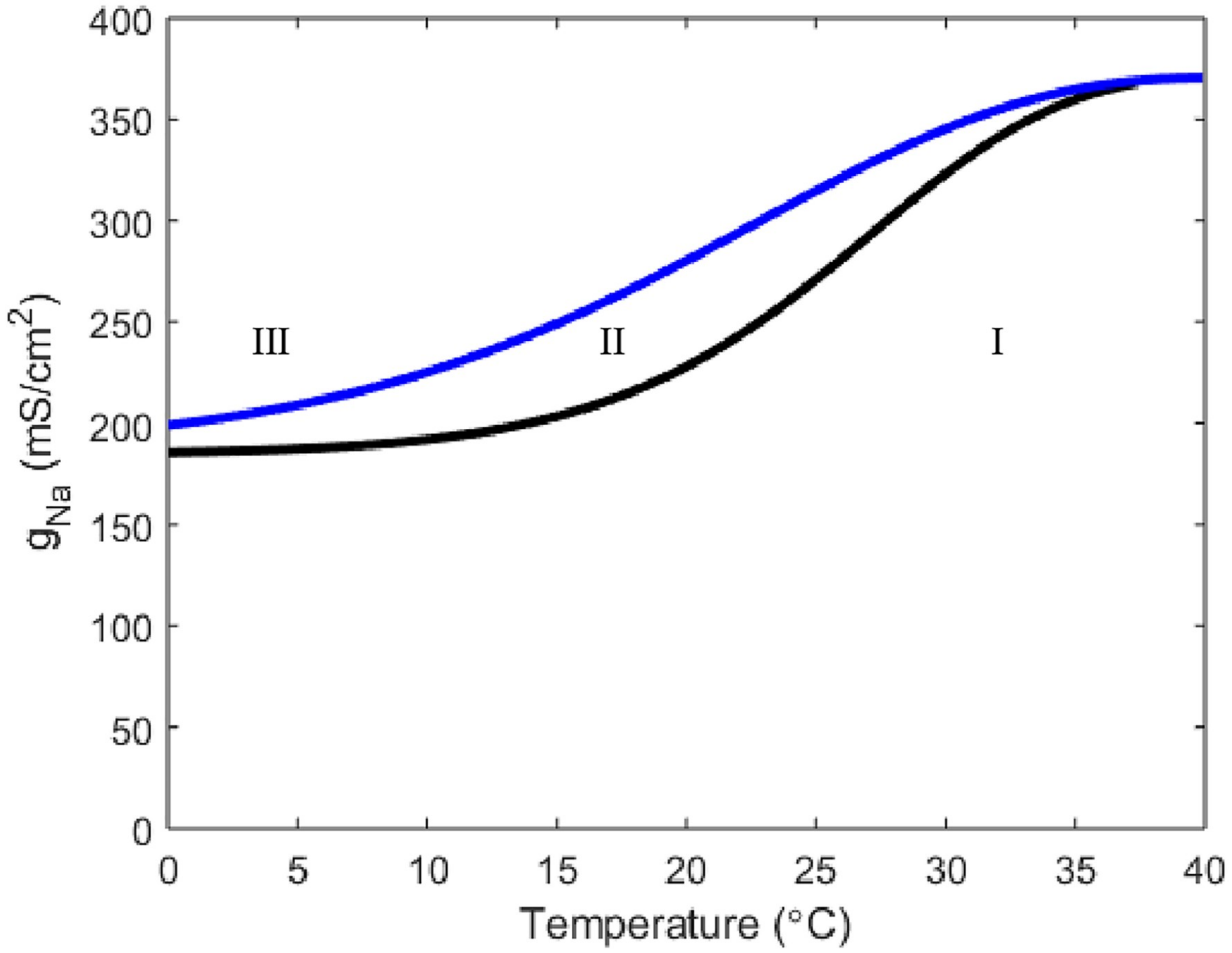
A two parameter bifurcation detailing the relationship between sodium maximal conductance, *g*_*Na*_ and temperature. The curve color and regions drawn are the same in meaning to those from Fig 1. Here *g*_*K*_ = 36 and *g*_*l*_ = .3.

The Hodgkin-Huxley framework also allows for an exploration of the ionic currents that haven’t been previously explicitly stated to have roles in threshold determination. If we adjust the leak current maximal conductance *g*_*l*_ but keep *g*_*K*_ and *g*_*Na*_ at the Hodgkin-Huxley values, no temperature dependence can be found. Alternatively, an increase of the sodium current maximal conductance can give rise to temperature responsiveness (Fig 3).

While there exist oscillatory regimes in Fig 3, they don’t arise unless *g*_*Na*_ is increased well above the Hodgkin-Huxley physiological value of *g*_*Na*_ = 120. Furthermore, at the first value for *g*_*Na*_ where periodic solutions arise, the oscillations only result from crossing a SNP, meaning depending on initial data the solution could be oscillatory or at stable steady state resting potential. Once *g*_*Na*_ reaches close to 200 at extreme low temperatures the neuron has crossed the Hopf bifurcation and into a region where the only stable solution is oscillatory.

**Fig 4:**
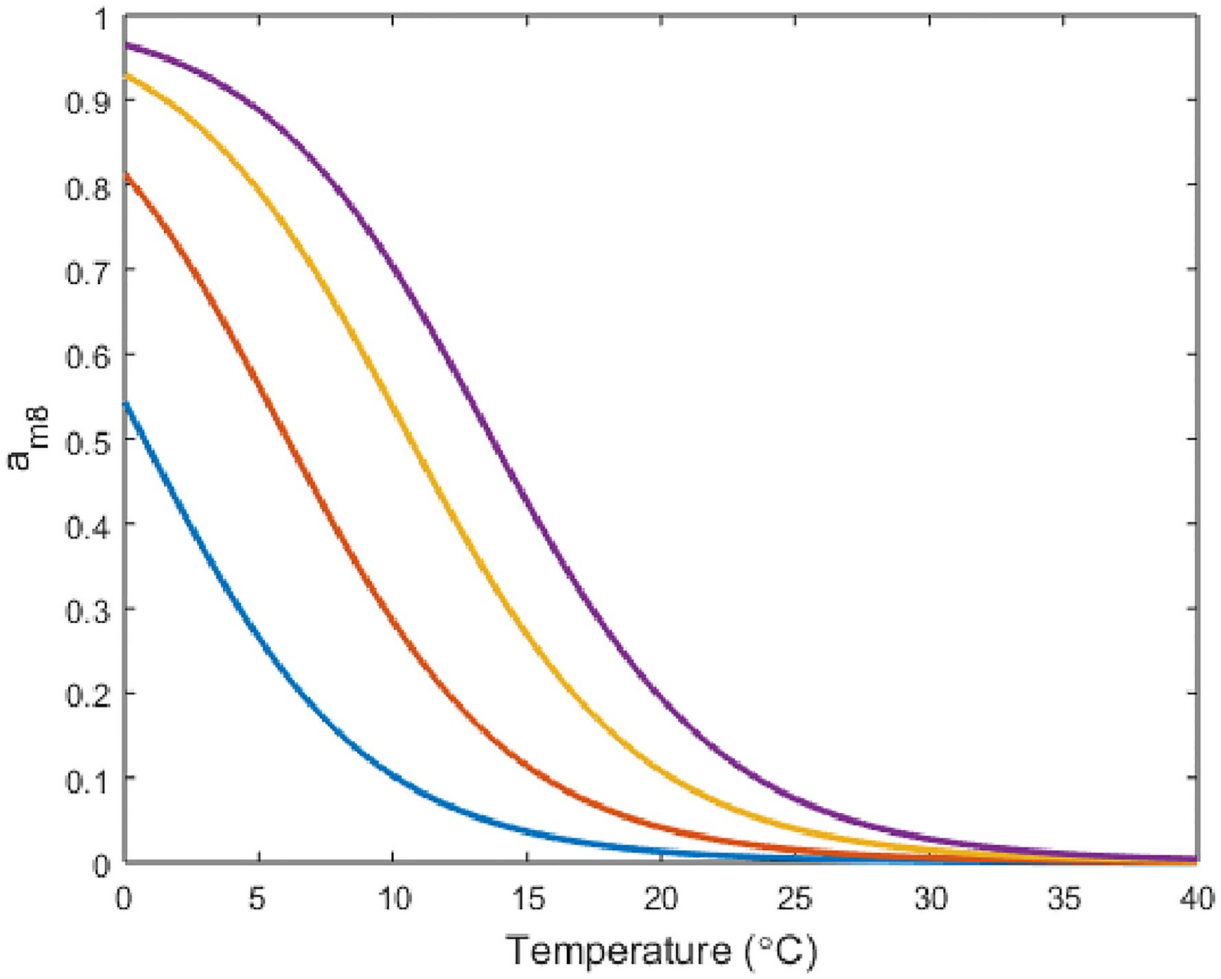
TRPM8 channel open probability plotted in response to temperature. Curve color corresponds to different membrane potential levels of -65mV(blue), -30mV(red), 0mV(yellow) and 20mV(purple).

**Fig 5:**
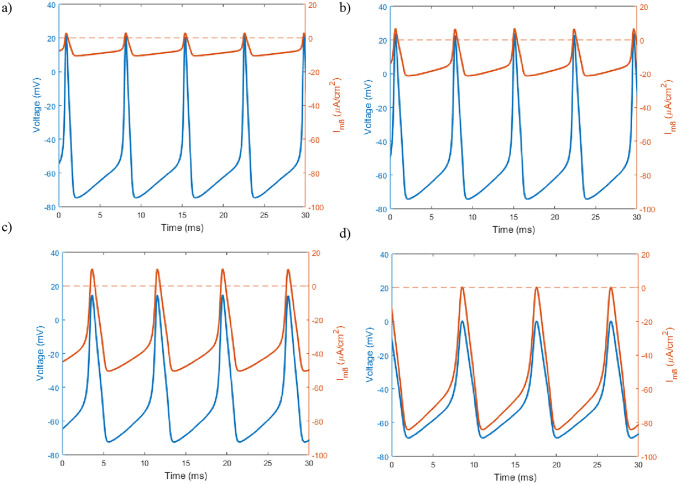
Sample traces of the neuron’s membrane potential and TRPM8 current in response to different static temperature levels. a-d) Temperature is set to 15°, 12°, 8° and 5° Celsius respectively. The membrane voltage is in blue with the TRPM8 current overlaid in red. The red dashed line denotes where the TRPM8 current switches from inward (negative values) to outward (positive values). Here *g*_*m*8_ = 3 and all other parameter values are at set to Hodgkin-Huxley standards.

Next, we add in the TRPM8 component of the model by making *g*_*m*8_ nonzero. We begin by showing a plot of the open probability of TRPM8 channels, *a*_*m*8_, in response to temperature at different physiological voltages (Fig 4). Fig 4 gives a rough idea of how the TRPM8 current will respond to temperature and voltage which combined with the voltage simulation in Fig 5 helps describe the neuron’s overall behavior at different temperature levels.

**Fig 6:**
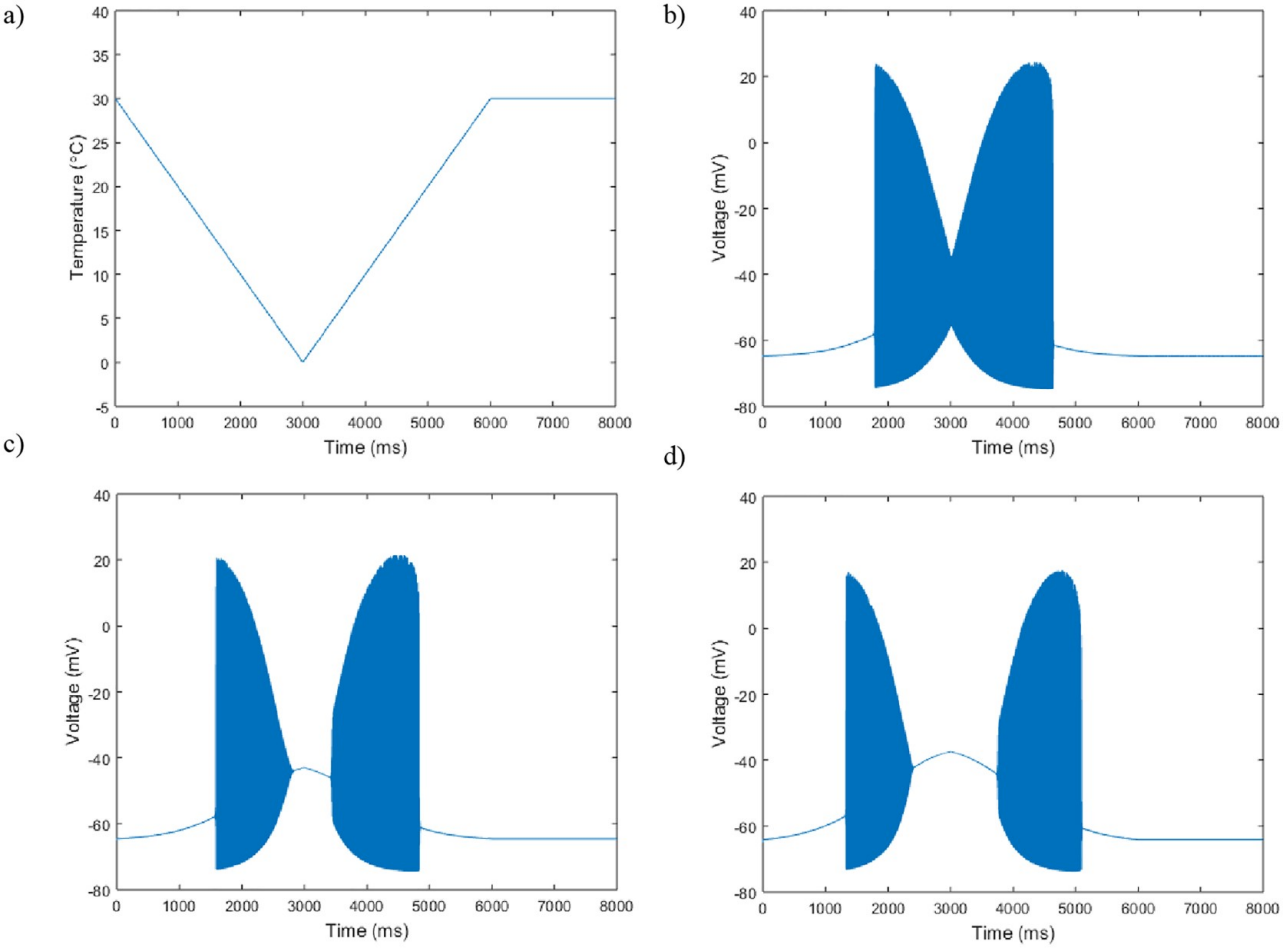
Membrane potential plotted against time in response to a simulated temperature ramp. a) The simulated change in temperature over time. b) The neuron’s periodic spiking behavior response to the temperature change plotted against time with *g*_*m*8_ = 3. c) Here *g*_*m*8_ = 5. d) Here *g*_*m*8_ = 10. All other parameter values are set to Hodgkin-Huxley values.

From Fig 5 a few noticeable features arise as the fixed temperature level is decreased. With decreasing temperatures the simulation reveals decreasing amplitudes of the action potentials as a result of decreasing the maximum voltage attained (Fig 5). Additionally, the overall amplitude of the TRPM8 current continues to grow with decreasing temperatures. We note that since *E*_*m*8_ = 0 this allows for the TRPM8 current to be both inward and outward depending on the membrane potential. The change in TRPM8 current amplitude is mainly due to the increase in inward flux, although it is noteworthy that at 8°C and 12°C there’s an increase in outward current. It is also informative to examine the behavior as *g*_*m*8_ is increased in response to a simulated temperature ramp (Fig 6).

Across the three different values of *g*_*m*8_ there are two consistent features, and one that arises as *g*_*m*8_ is increased. In all three plots there are large amplitude jumps in the oscillations when the neuron first turns on and when it turns off. Furthermore, in each of the three plots, the neuron’s activity on the down ramp and up ramp of temperature appear asymmetric, with the oscillations on the increasing temperature ramp lasting longer. Finally, as *g*_*m*8_ is increased, the neuron ceases to have oscillations in the coldest temperature portion of the ramp. The plots with *g*_*m*8_ = 5 and *g*_*m*8_ = 10 also show how the oscillations shrink in amplitude as the neuron is exposed to decreasing temperatures.

**Fig 7:**
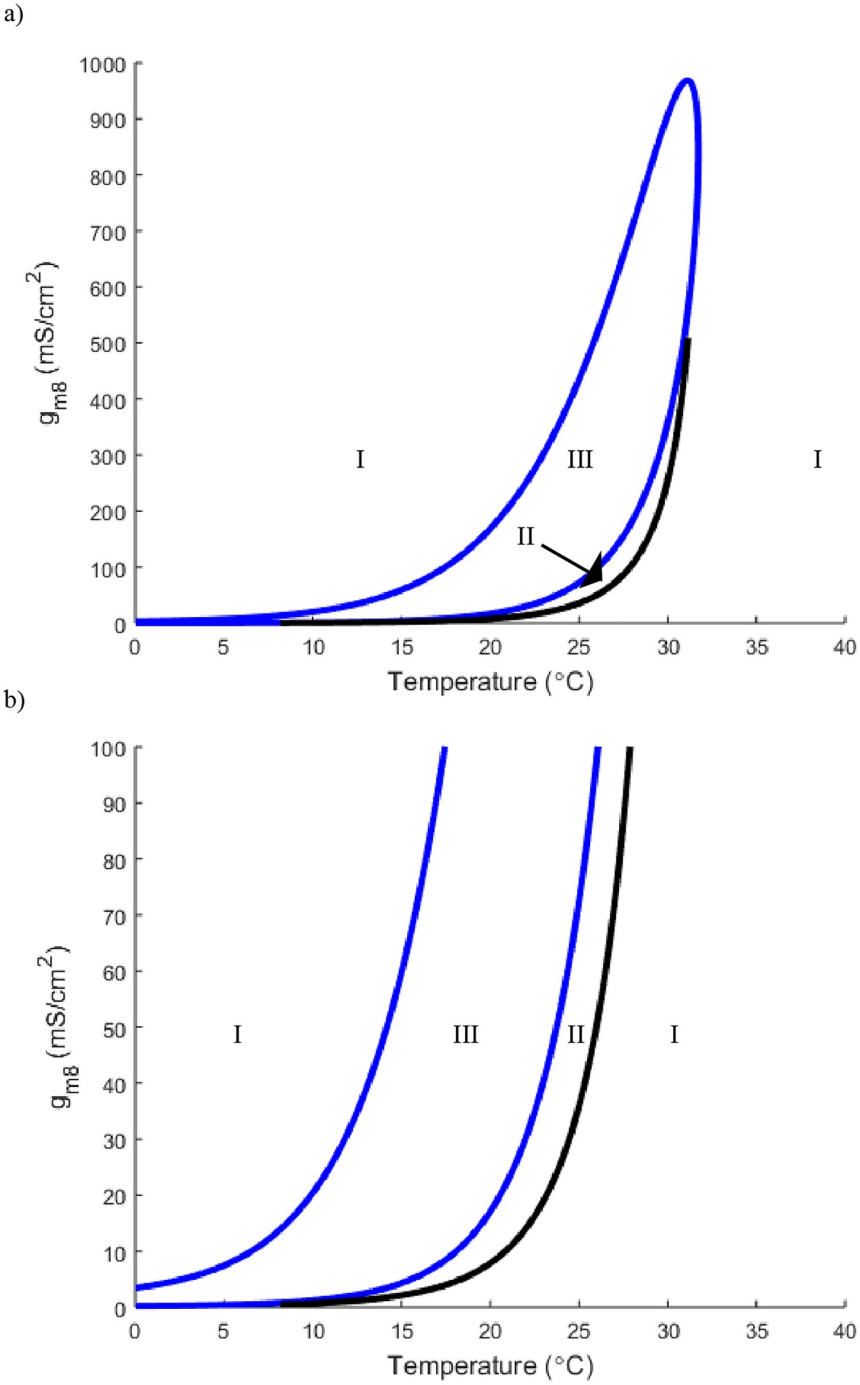
a) A two parameter bifurcation demonstrating the relationship between temperature and *g*_*m*8_. b) a zoomed in version of Fig 6a. As in Fig 1, The blue curve represents the Hopf bifurcation while the black curve is a saddle-node-periodic (SNP) bifurcation. The regions are labeled as in Fig 1. Here *g*_*K*_ is set to 36.

**Fig 8:**
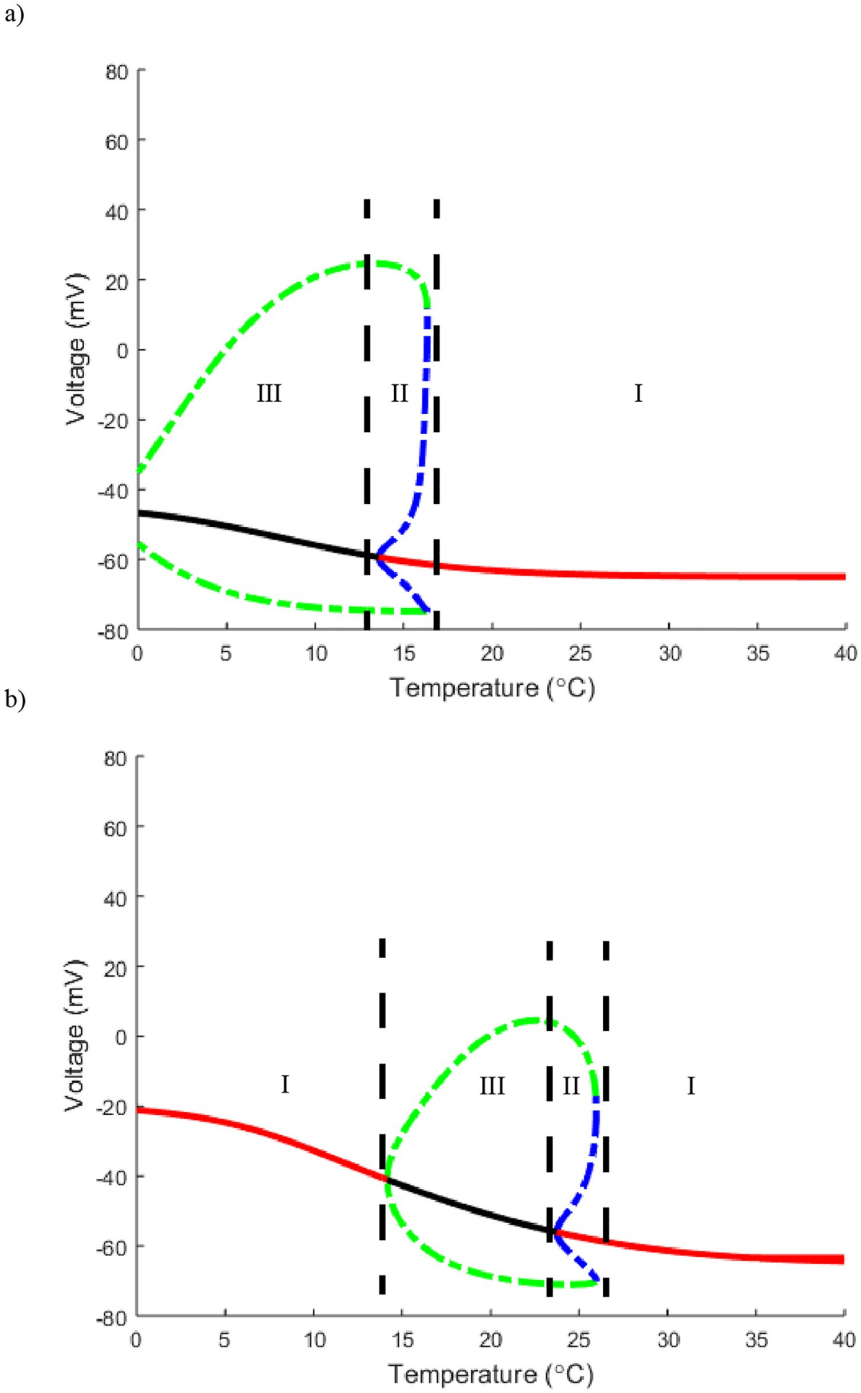
Horizontal one parameter bifurcation slices of Fig 6. a) A slice with *g*_*m*8_ set to 3. b) A horizontal slice with *g*_*m*8_ set to 50. In both a) and b) *g*_*K*_ = 36. Curve color and labeled regions are identical in meaning to those in Fig 2.

Although these Voltage-Time plots give a general idea of the effect that TRPM8 channels have on the neuron’s activation and inactivation, we wish to give fuller explanation of the interplay between each of the ionic currents and temperature. In Fig 7 we focus on *g*_*m*8_ and its relationship with temperature in a two parameter bifurcation diagram. Critically, with *g*_*K*_ = 36, the standard Hodgkin-Huxley value, starting in region I on the right (30°C) and decreasing temperature turns on the neuron by crossing through the SNP bifurcation and then the Hopf bifurcation. We highlight the importance of the SNP bifurcation and region II in general as the existence of the unstable oscillatory solution implies that a slight perturbation or input could turn the neuron on from the stable steady state. Looking at horizontal slices of this two parameter bifurcation in Fig 7 we easily see the shift of the temperature threshold. Changing *g*_*m*8_ = 3 to *g*_*m*8_ = 50 from Fig 8a to 8b changes how much less temperature must drop to turn the neuron on with higher *g*_*m*8_ values.

**Fig 9:**
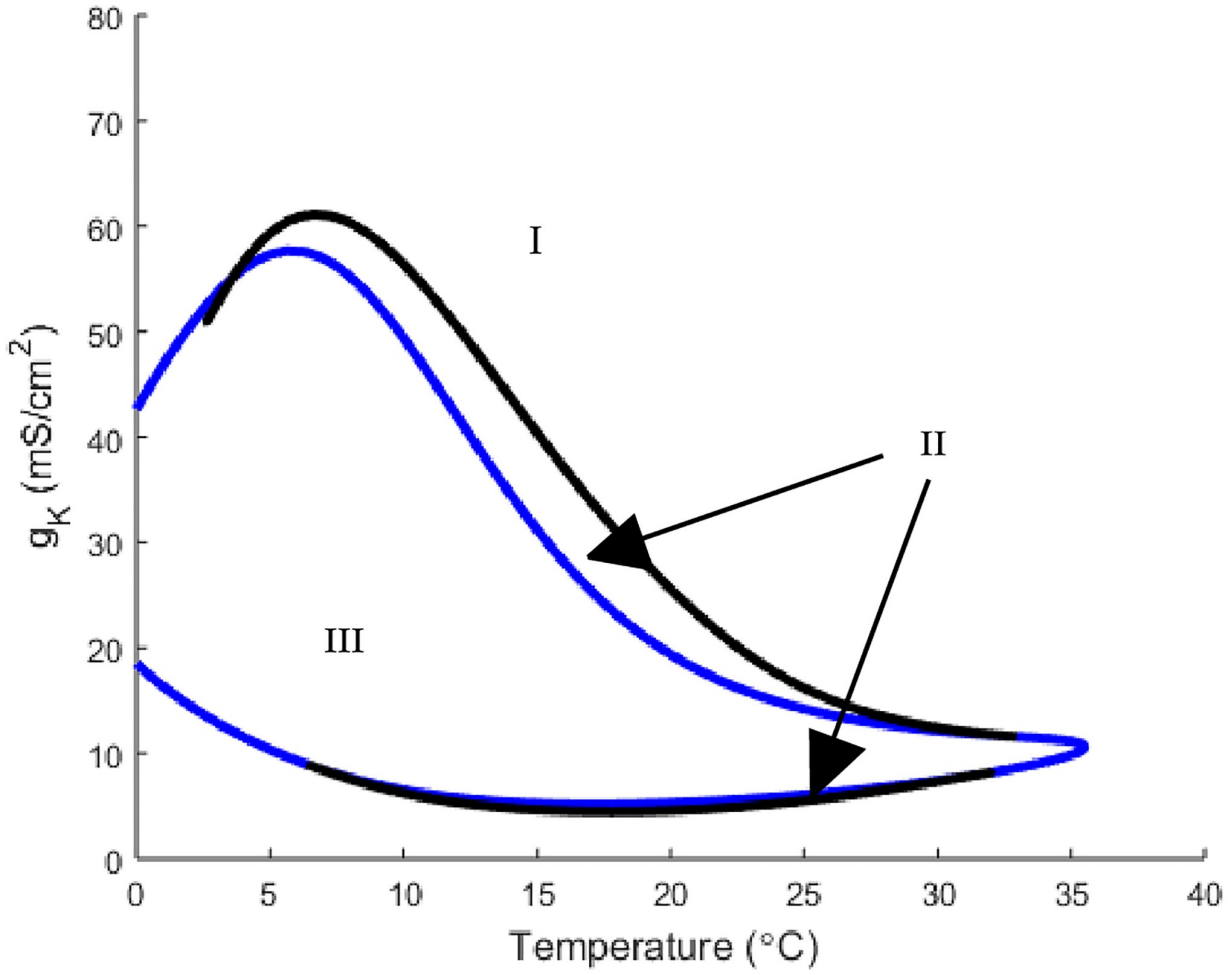
A two parameter bifurcation of the relationship between temperature and *g*_*K*_. Curve color and labeled regions are identical in meaning to those in Fig 1. Here *g*_*m*8_ = 3.

**Fig 10:**
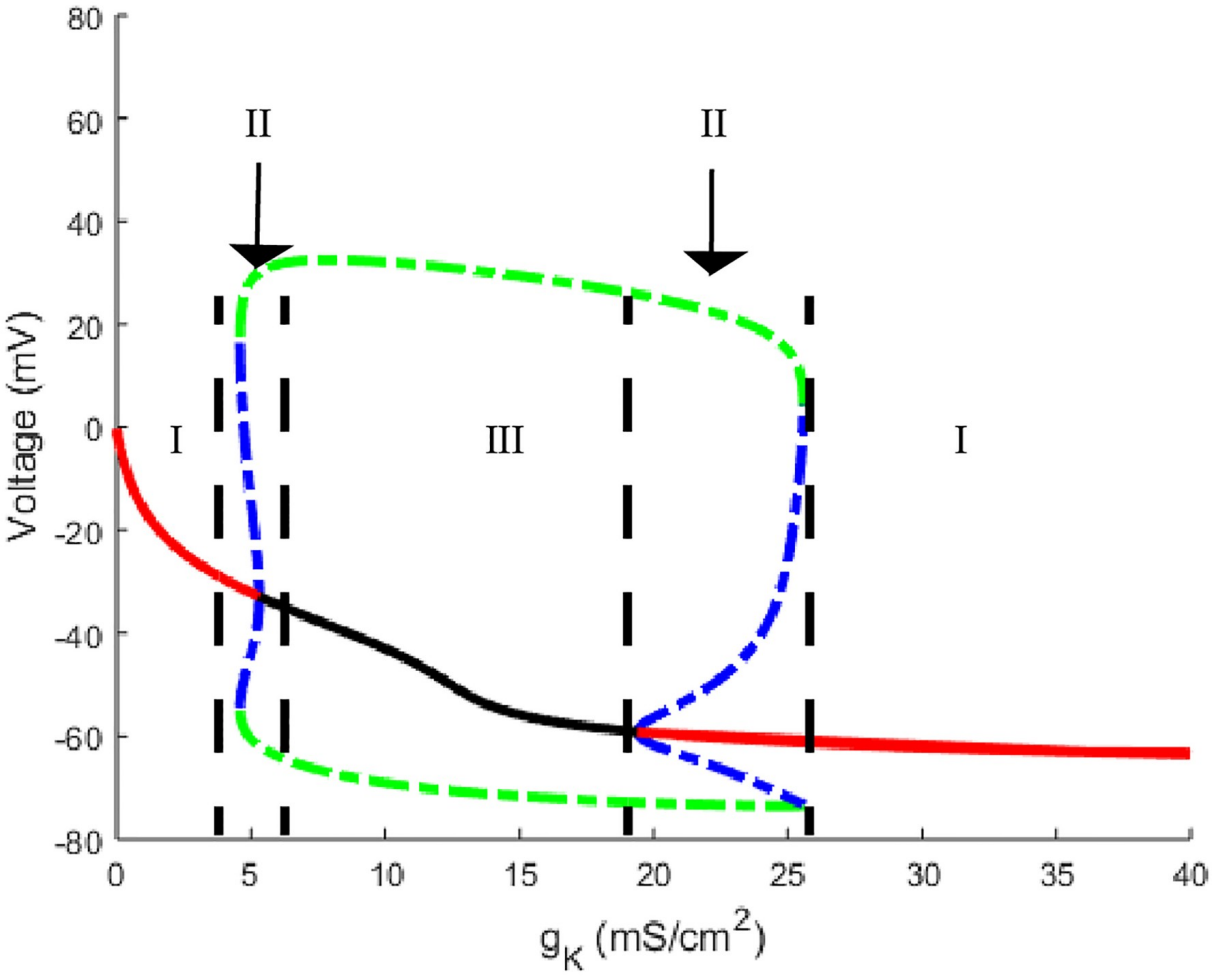
A horizontal slice from Fig 8. Temperature is set to 20 degrees Celsius and *g*_*m*8_ is set to 3. Curve color and labeled regions are identical in meaning to those in Fig 2.

Returning to the high versus low threshold phenomenon, keeping otherwise standard Hodgkin-Huxley parameter values, increasing *g*_*m*8_ shifts the neuron from a higher to a lower activation threshold (Figs 7 and 8). Additionally, with *g*_*m*8_ = 3 we can consider traveling horizontally through Fig 9 at values *g*_*K*_ = 45 and *g*_*K*_ = 20. This shows that decreasing *g*_*K*_ shifts the Hopf and SNP curves further right thus requiring a lower drop in temperature in order to turn on. A representative vertical slice in Fig 10 showcases the transitions from stable steady state solution to oscillatory and back.

**Fig 11:**
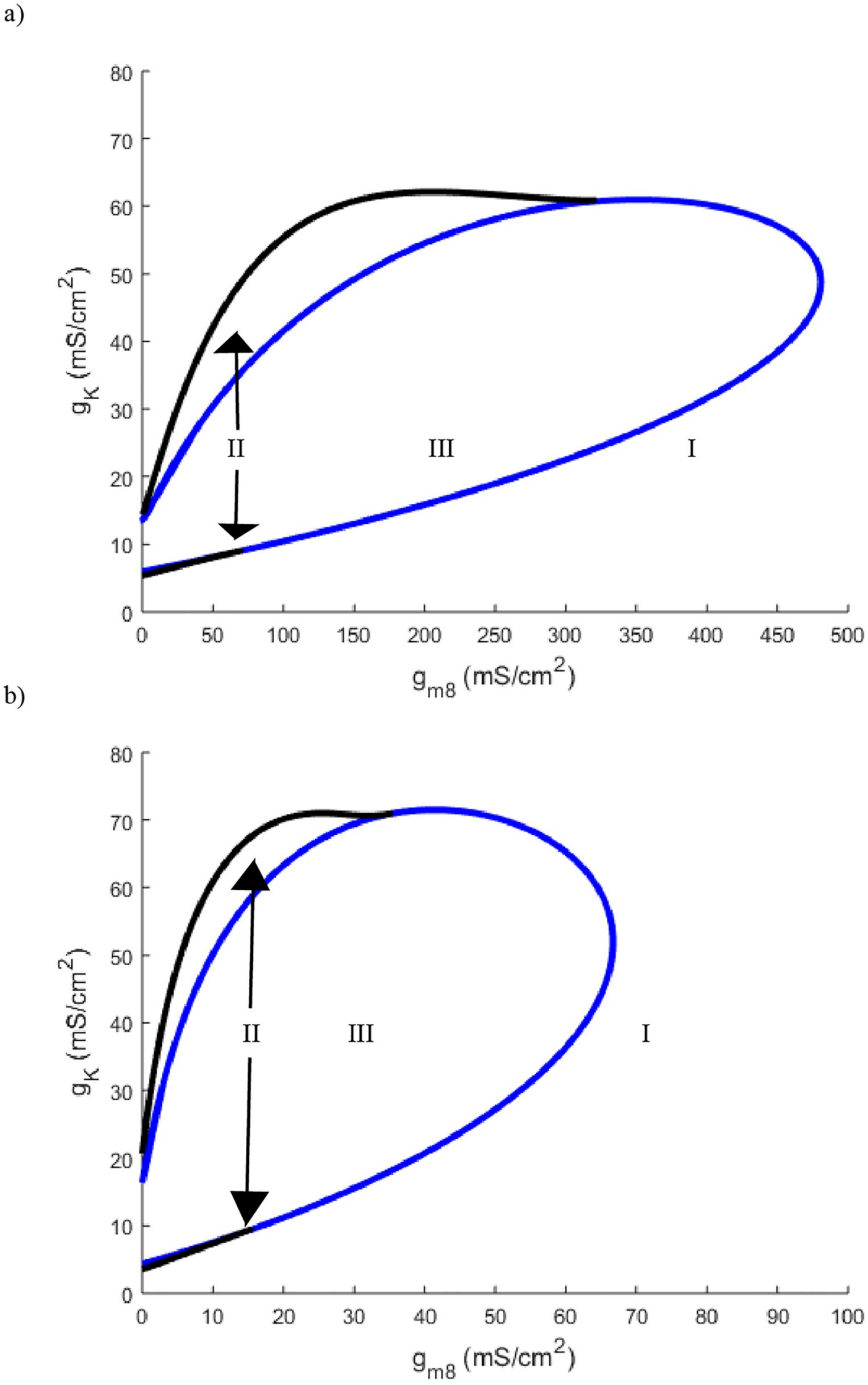
a) Two parameter bifurcation of *g*_*m*8_ plotted against *g*_*K*_ with temperature set to 25°C. b) Two parameter bifurcation of *g*_*m*8_ plotted against *g*_*K*_ with temperature set to 15°C. Curve color and labeled regions are identical in meaning to those in Fig 1.

**Fig 12:**
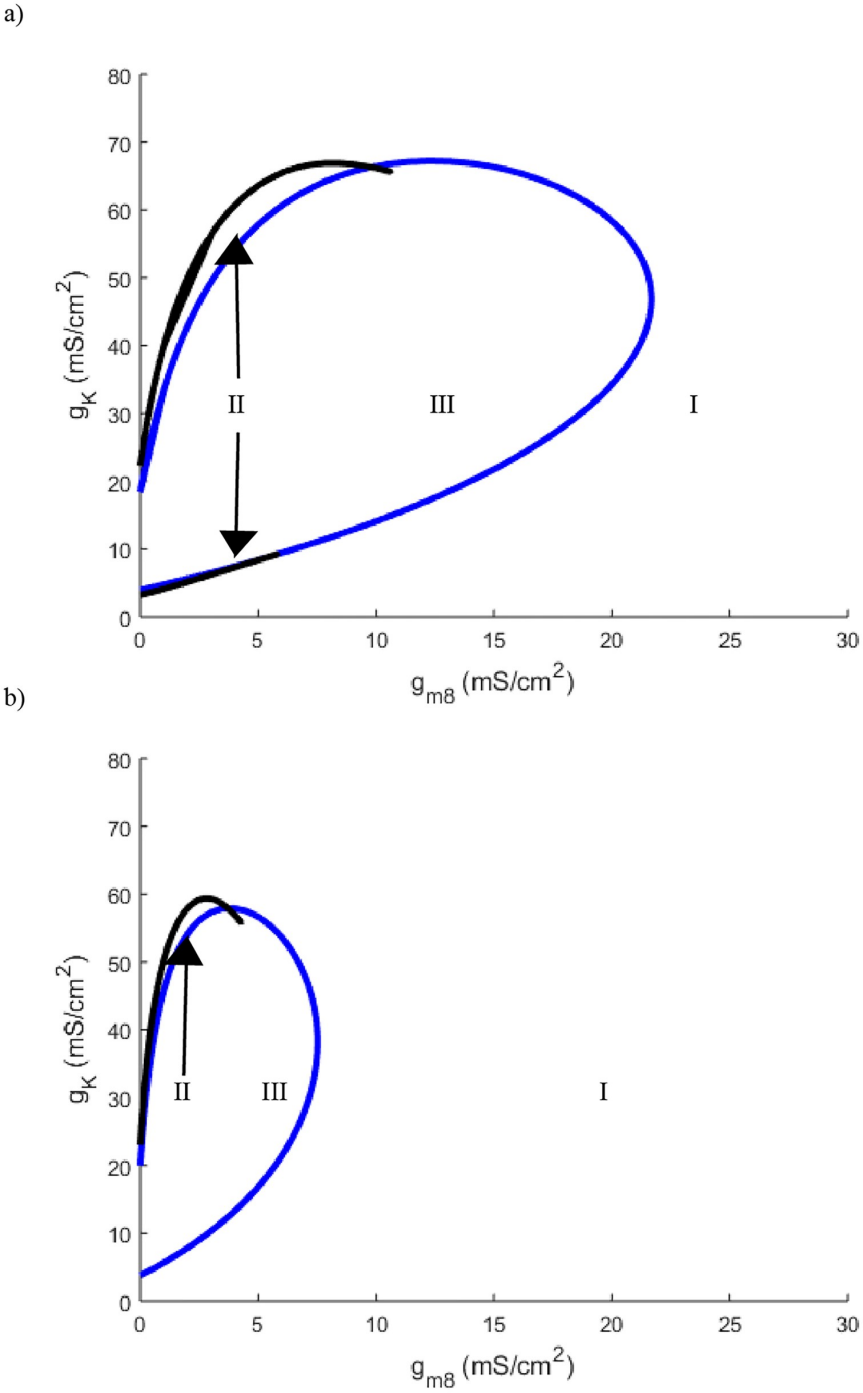
a) Two parameter bifurcation of *g*_*m*8_ plotted against *g*_*K*_ with temperature set to 10°C. b) Two parameter bifurcation of *g*_*m*8_ plotted against *g*_*K*_ with temperature set to 5°C. Curve color and labeled regions are identical in meaning to those in Fig 1.

To summarize the relationship between TRPM8 and voltage gated potassium channels, plots of *g*_*m*8_ against *g*_*K*_ at four different temperature values are provided in Figs 11 and 12. The first observation is that the total area of parameter space in which there are oscillations decreases as temperature decreases. Yet in spite of this, each plot has the feature that starting in region III, where there are oscillatory solutions, and decreasing *g*_*m*8_ takes the neuron to region I where there are no periodic solutions. This can then be counterbalanced by decreasing *g*_*K*_ to take the neuron back into region III. Similar transitions between regions takes place if *g*_*K*_ is decreased first followed by a decrease in *g*_*m*8_ (Figs 11 and 12).

**Fig 13:**
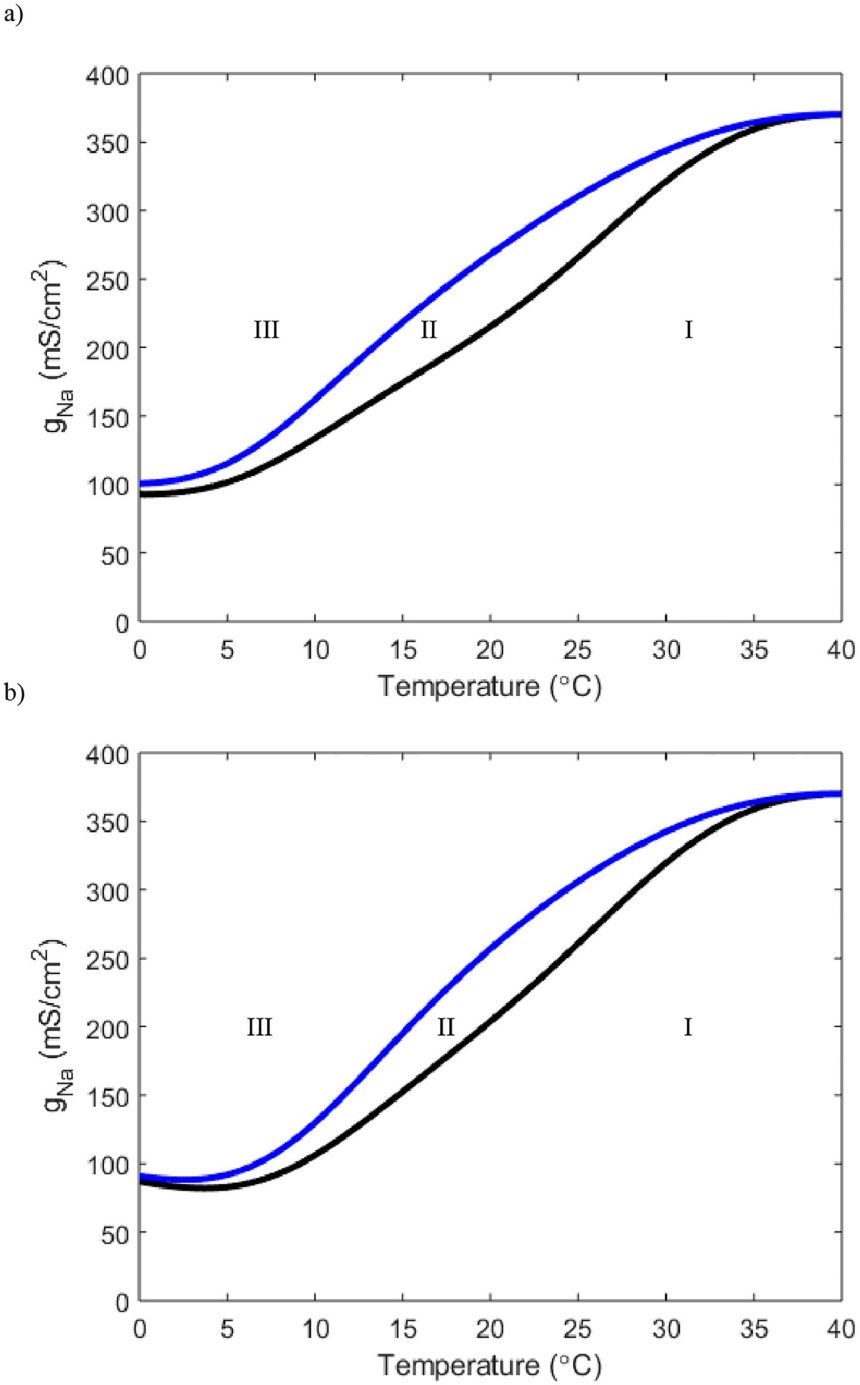
Two parameter bifurcations showing the relationship between temperature and *g*_*Na*_. In the top plot *g*_*m*8_ = .5 and in the bottom *g*_*m*8_ = 1. Both *g*_*l*_ and *g*_*K*_ are set to Hodgkin-Huxley values. Curve color and region meaning is equivalent to other two parameter bifurcation plots.

With a clearer idea of how the TRPM8 current interacts with temperature and the potassium current to determine the threshold level we turn to the sodium and leak currents. By increasing *g*_*m*8_ we can see the relationship temperature has with *g*_*Na*_ (Fig 13).

**Fig 14:**
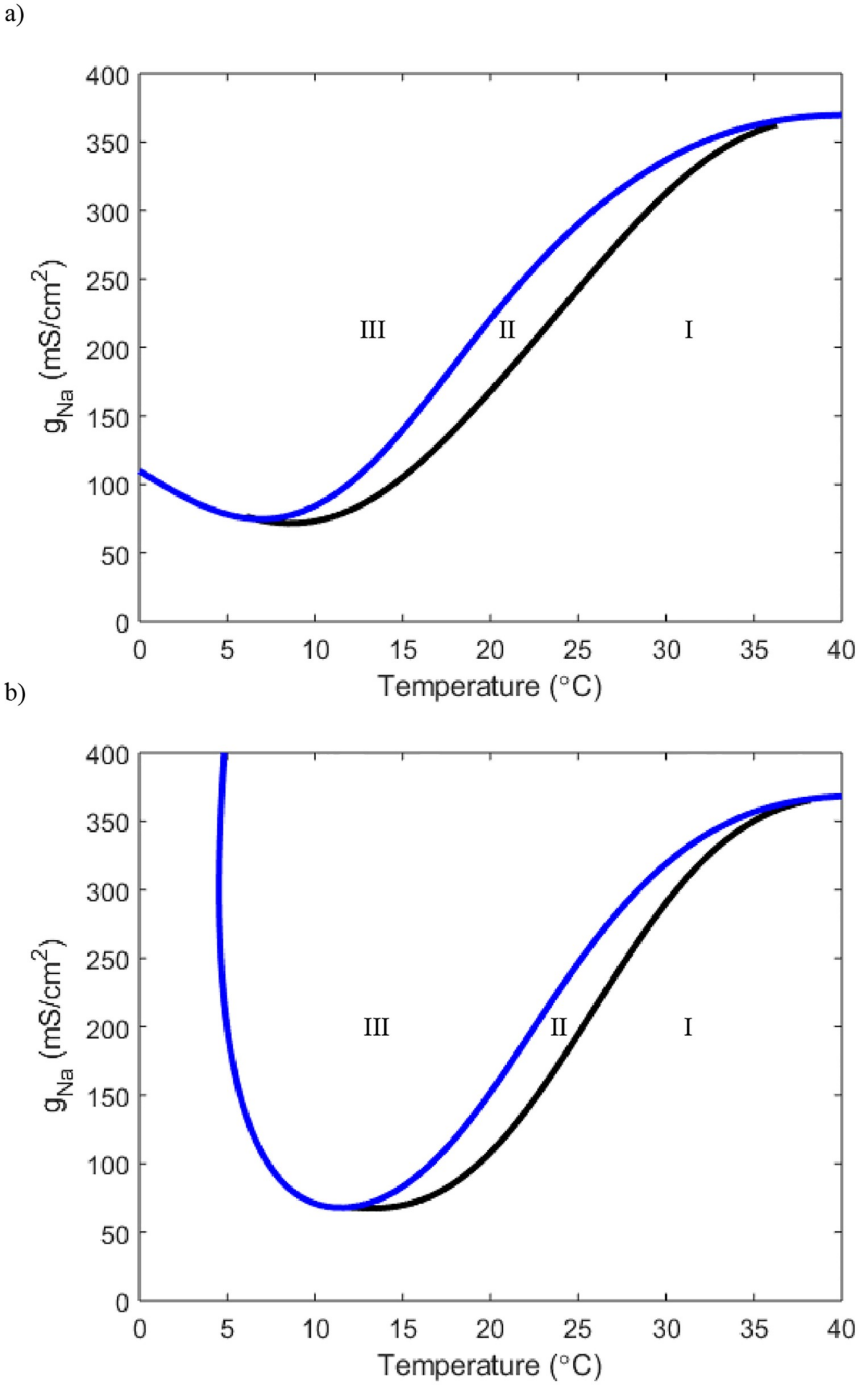
Two parameter bifurcations showing the relationship between temperature and *g*_*Na*_. In the top plot *g*_*m*8_ = 3 and in the bottom *g*_*m*8_ = 10. Both *g*_*l*_ and *g*_*K*_ are set to Hodgkin-Huxley values. Curve color and region meaning is equivalent to those in Fig 1.

At these low of values of *g*_*m*8_ we see similar behavior to Fig 3. Of note, the neuron now does turn on in the horizontal slice of *g*_*Na*_ = 120, but this still occurs only at extreme low temperatures. Furthermore we still see that for a tiny window of *g*_*Na*_ values we can obtain periodic solutions that are only a result of the SNP. Yet if we increment *g*_*m*8_ further we can see temperature specific windows of periodic solutions arise (Fig 14).

**Fig 15:**
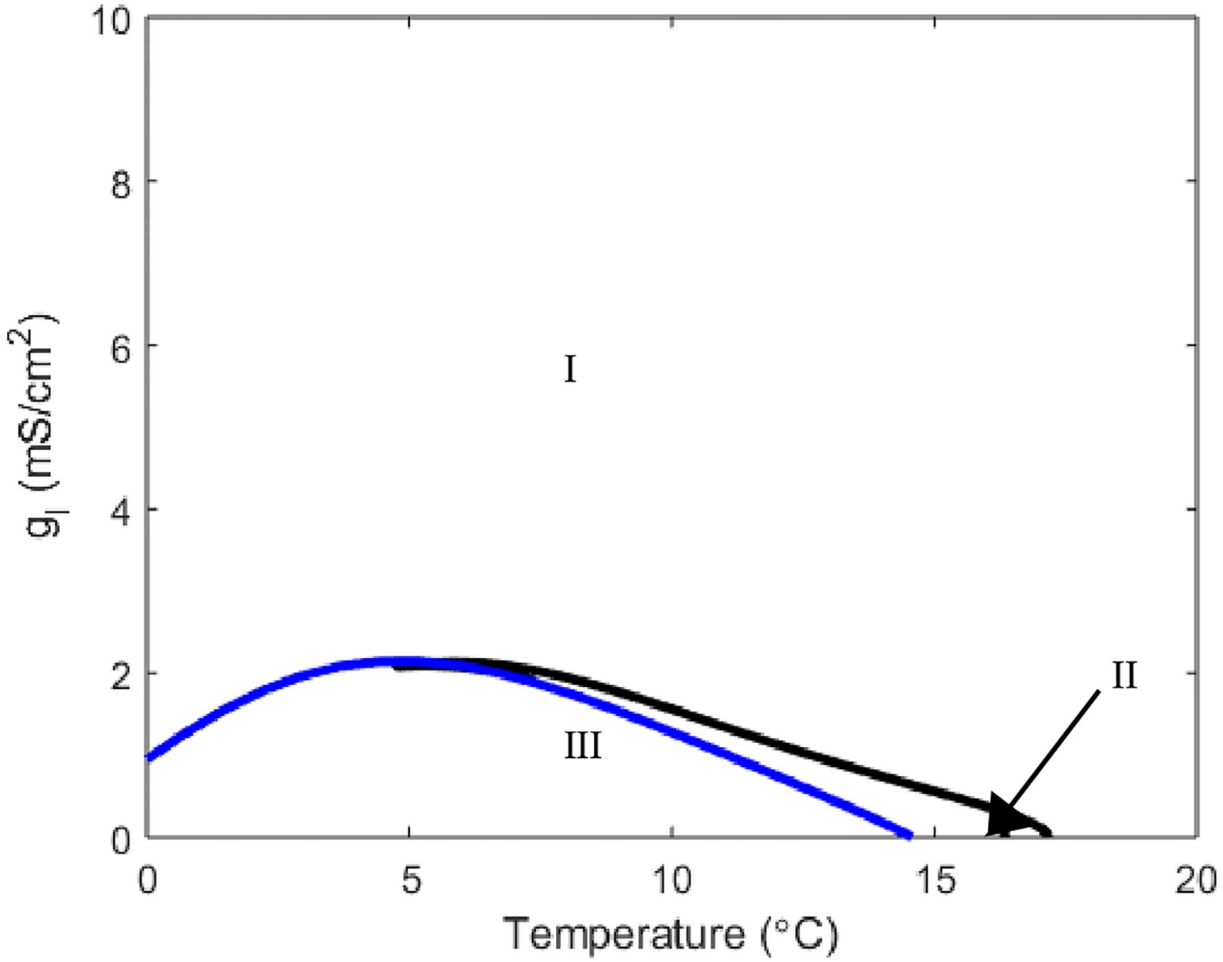
Two parameter bifurcation plot relating *g*_*l*_ to temperature. Here *g*_*m*8_ = 3. All other parameters are set to Hodgkin-Huxley value with curve color and region meaning equivalent to those in Fig 1.

We see that with *g*_*m*8_ = 3, at values for *g*_*Na*_ between 60 and 100, horizontal slices result in the neuron turning on and off again at specific temperatures. Further emphasis of how these neurons’ temperature responses can be finely tuned is shown for *g*_*m*8_ = 10. Horizontal slices at different *g*_*Na*_ values through Fig 14b reveal temperature windows of different lengths and of different intervals. A similar observation can be made about the effect the leak current has on the neuron’s degree of response (Fig 15).

Taking a horizontal slice with *g*_*l*_ = .3 as it is in the canonical Hodgkin-Huxley formulation we have temperature sensitivity. Yet as with the sodium current, we can tune the width of temperature range in which this neuron is active by increasing *g*_*l*_.

## Discussion

As advancements are made in the field of neuroscience, labs are beginning to examine properties of a variety of different neuron classes. One of the classes that garnered some interest recently was cold thermosensors. Particular attention was given to the role of their ion channels, critically the TRPM8 and voltage gated potassium channels. Here we have developed a simple model of a cold thermosensor centered around a Hodgkin-Huxley base with an added TRPM8 component. The goal here was to create a physiological model that would allow for investigation into the roles and properties of the involved ion channels and how they interact with changes in temperature.

The first point we emphasize is that the Hodgkin-Huxley model at standard parameter values does not have sufficient temperature dependence to account for the known data. Adding a TRPM8 channel to the model results in the appearance of oscillatory solutions for the neuron with temperature decrease (Figs 8 and 9). This coincides with lab findings highlighting the importance of TRPM8 channels within the class of cold thermosensors [2, 4, 5]. Previous work also highlights the variety of cold sensing neurons describing a range from high to low threshold neurons with a range of activation temperatures [3, 5]. One defining characteristic that separated high from low threshold was found to be the effectiveness of the TRPM8 channels [2, 8]. With a higher density of TRPM8 channels, the neuron can activate at higher temperature values [8]. Our model has this same graded temperature threshold showing that a change in maximal conductance of TRPM8 channels changes how far temperature must drop to obtain an oscillatory solution. Specifically, we show that at otherwise standard Hodgkin-Huxley parameter values with *g*_*m*8_ = 3 the temperature threshold is 15°*C* to turn on while at *g*_*m*8_ = 50 the threshold is 25°*C* (Figs 7 and 8).

We also demonstrate what the oscillation profiles look like in response to different temperature levels. We show how the changes in the neuron’s behavior is dependent on the growth of the TRPM8 inward current (Fig 5). The temperature ramp simulation with increasing TRPM8 maximal conductance helps give a clearer view of the response of what the neurons are physiologically exposed to (Fig 6). Figs 5 and 6 help provide insight into the cold receptor’s activation and inactivation in response to increasing and decreasing temperatures.

Although TRPM8 channels were identified as a main determining factor in the identification of cold thermosensors, many labs also highlight the importance of voltage gated potassium channels [2, 8]. While previous mathematical models of cold sensors had the desired response to the lowering of temperature, they lacked the ability to track specific ionic currents [10]. With the Hodgkin-Huxley model as a basis for our model, the potassium flux was able to be explicitly tracked. Altering the potassium channel maximal conductance also found the model to be in agreement with previous lab findings. Lowering the maximal conductance of potassium channels *g*_*K*_, can transition the neuron from high to low threshold (Fig 9). Also shown in lab settings, TRPM8 and voltage gated potassium channels have offsetting effects [8]. If TRPM8 maximal conductance is lowered, the neuron can be made non-responsive to temperature as seen in Figs 11 and 12 by going from region III to I. This can then be reversed by decreasing the potassium maximal conductance which takes the neuron back into region III. We note that this counterbalancing effect stems primarily from *g*_*K*_ being an outward current with *g*_*m*8_ being a largely inward current due to their reversal potentials. Thus this offsetting affect may be a general feature due more to the ion channel current directions than to specific ion channel type.

Our modelling framework simultaneously allows for exploration of the effects of the sodium and leak currents. The sodium current does not appear to have a role in setting the threshold level. For the sodium current, with *g*_*m*8_ = 0, the neuron will only activate in the extreme temperature range and not until *g*_*Na*_ is drastically increased away from Hodgkin-Huxley value (Fig 3). We also notice that not until *g*_*m*8_ = 3 does the neuron have stable oscillatory solutions from crossing a Hopf bifurcation point near values of *g*_*Na*_ = 120 (Figs 13 and 14). Although the sodium current does not appear to heavily dictate the threshold level for activation, it appears to have some control over the range of temperature the neuron can sense (Figs 13 and 14). After *g*_*m*8_ is increased above 3, we see that different horizontal slices yield different temperature windows of activation and inactivation (Fig 14b). This implies that the specificity of exactly when the cold thermosensors turn on and off may be modulated by the number of sodium channels present.

This temperature window feature is also observable with alterations to the leak current maximal conductance. In particular with *g*_*l*_ = .3 as it is in Hodgkin-Huxley and *g*_*m*8_ = 3 we see the neuron turns on and stays on past 0°C (Fig 15). Yet increasing *g*_*l*_ to be between 1 and 2 we can see that for a horizontal slice we have a temperature value in which oscillatory solutions arise and then a second temperature before 0°C where the neuron returns to a stable steady state solution (Fig 15). The analysis of the sodium and leak currents indicates while the main threshold level is set by the relationship between the TRPM8 and potassium currents, these currents may still have a role to play. Finely tuned temperature responses may be dictated by the strength of the sodium and leak currents.

Finally, while the majority of previous work centered upon investigating at what point these cold sensing neurons turn on, this model provides additional insight into the neuron’s inactivation. Fig 4 shows the TRPM8 current is activated at these lower temperature for all relevant voltages. However, in Figs 5 and 6, we can see the full behavior of the neuron at these lower temperatures, with a decrease in spiking amplitude height corresponding to an increase in inward TRPM8 current. From Fig 7 we see that if *g*_*m*8_ is fixed at any value near or above 10, lowering temperature far enough will inactivate the neuron by returning it to region I. In particular, as seen in Fig 8b, region I on the left corresponds to only a single stable steady state. However, it is important to note that this steady state is at a much higher resting membrane voltage, -20mV, than the typical resting potential range of -65mV. This observation lends itself to the idea that either at extremely low temperatures the neuron is overstimulated and can no longer fire action potentials, or the current model does not include enough ion channels to fully encapsulate the behavior of cold sensing neurons in extreme temperatures.

We see a similar phenomenon at extreme low temperatures with regards to lowering *g*_*K*_. In the temperature window of 0° *C*-10° *C*, decreasing *g*_*K*_ causes the neuron to transition to region I with only a single steady state (Fig 9). As was seen in Fig 8 when altering *g*_*m*8_, by decreasing *g*_*K*_ far enough the neuron is depolarized with a single steady state but at a much higher value, near -20mV, than a standard Hodgkin-Huxley neuron resting potential (Fig 10). These observations are corroborated by Figs 11 and 12 which highlight that as temperature decreases towards the extreme, the total area of region III decreases drastically. From this we can hypothesize that either the cold sensitive neurons at extremely low temperatures are extremely specific as to the densities of their ion channels, or as predicted by some, there are other channels at play including the TTX resistant *Na*_*V*_1.8 and possibly TRPVA1 channels [5, 8, 16].

## Conclusion

The general Hodgkin-Huxley structure of this model meant that specific ionic currents, could be tracked. Unlike in previous cold sensing neuron models where the types of ionic currents were not explicit, this new model allows for examination of high versus low threshold neurons and the specific temperature levels of activation and inactivation. We demonstrated the value an in-depth bifurcation analysis can provide for answering biological questions. This analysis helped solidify some of the primary features of cold sensing neurons: the presence of TRPM8 channels, different threshold neurons and the interplay between the TRPM8 current and the voltage gated potassium current. Additionally, the analysis showed that the leak and sodium currents could act as mediators of the temperature window in which these neurons activate. By starting with a physiological basis and then adding the highlighted current TRPM8, we were able to provide an improved overall understanding of the neuron class of cold thermosensors.
