# Supplementary material for: A mathematical model analyzing temperature threshold dependence in cold sensitive neurons
Source: PLoS One. 2020 Aug 12;15(8):e0237347. doi: 10.1371/journal.pone.0237347 (PMC7423123; doi:10.1371/journal.pone.0237347)
Supplement: Peer review history

# Peer review: Decision Letter 0

**DOI:** 10.1371/journal.pone.0237347.r001

**Authors:** David D McKemy

5 May 2020

PONE-D-20-10272

A mathematical model analyzing temperature threshold dependence in cold sensitive neurons

PLOS ONE

Dear Mr. McGahan,

Thank you for submitting your manuscript to PLOS ONE. After careful consideration, we feel that it has merit but does not fully meet PLOS ONE’s publication criteria as it currently stands. Therefore, we invite you to submit a revised version of the manuscript that addresses the points raised during the review process.

We would appreciate receiving your revised manuscript by Jun 19 2020 11:59PM. When you are ready to submit your revision, log on to https://www.editorialmanager.com/pone/ and select the 'Submissions Needing Revision' folder to locate your manuscript file.

If you would like to make changes to your financial disclosure, please include your updated statement in your cover letter.

To enhance the reproducibility of your results, we recommend that if applicable you deposit your laboratory protocols in protocols.io, where a protocol can be assigned its own identifier (DOI) such that it can be cited independently in the future. For instructions see: http://journals.plos.org/plosone/s/submission-guidelines#loc-laboratory-protocols

Please include the following items when submitting your revised manuscript:

- A rebuttal letter that responds to each point raised by the academic editor and reviewer(s). This letter should be uploaded as separate file and labeled 'Response to Reviewers'.
- A marked-up copy of your manuscript that highlights changes made to the original version. This file should be uploaded as separate file and labeled 'Revised Manuscript with Track Changes'.
- An unmarked version of your revised paper without tracked changes. This file should be uploaded as separate file and labeled 'Manuscript'.

Please note while forming your response, if your article is accepted, you may have the opportunity to make the peer review history publicly available. The record will include editor decision letters (with reviews) and your responses to reviewer comments. If eligible, we will contact you to opt in or out.

We look forward to receiving your revised manuscript.

Kind regards,

David D McKemy

Academic Editor

PLOS ONE

Journal Requirements:

When submitting your revision, we need you to address these additional requirements.

1. Please ensure that your manuscript meets PLOS ONE's style requirements, including those for file naming. The PLOS ONE style templates can be found at https://journals.plos.org/plosone/s/file?id=wjVg/PLOSOne_formatting_sample_main_body.pdf and https://journals.plos.org/plosone/s/file?id=ba62/PLOSOne_formatting_sample_title_authors_affiliations.pdf

Additional Editor Comments (if provided):

[Note: HTML markup is below. Please do not edit.]

Reviewers' comments:

Reviewer's Responses to Questions

**Comments to the Author**

1. Is the manuscript technically sound, and do the data support the conclusions?

The manuscript must describe a technically sound piece of scientific research with data that supports the conclusions. Experiments must have been conducted rigorously, with appropriate controls, replication, and sample sizes. The conclusions must be drawn appropriately based on the data presented. 

Reviewer #1: No

Reviewer #2: Partly

**********

2. Has the statistical analysis been performed appropriately and rigorously? 

Reviewer #1: N/A

Reviewer #2: N/A

**********

3. Have the authors made all data underlying the findings in their manuscript fully available?

The PLOS Data policy requires authors to make all data underlying the findings described in their manuscript fully available without restriction, with rare exception (please refer to the Data Availability Statement in the manuscript PDF file). The data should be provided as part of the manuscript or its supporting information, or deposited to a public repository. For example, in addition to summary statistics, the data points behind means, medians and variance measures should be available. If there are restrictions on publicly sharing data—e.g. participant privacy or use of data from a third party—those must be specified.

Reviewer #1: No

Reviewer #2: No

**********

4. Is the manuscript presented in an intelligible fashion and written in standard English?

PLOS ONE does not copyedit accepted manuscripts, so the language in submitted articles must be clear, correct, and unambiguous. Any typographical or grammatical errors should be corrected at revision, so please note any specific errors here.

Reviewer #1: Yes

Reviewer #2: Yes

**********

5. Review Comments to the Author

Please use the space provided to explain your answers to the questions above. You may also include additional comments for the author, including concerns about dual publication, research ethics, or publication ethics. (Please upload your review as an attachment if it exceeds 20,000 characters)

Reviewer #1: The work presents a mathematical model to analyze the temperature dependence of the threshold of cold sensitive neurons.

The model is simple, consisting only of Hogking-Huxley conductances plus a model of TRPM8 channels that are temperature and voltage dependent.

The effect of TRPM8 channels on the activity of cold sensitive neurons has been modeled before and the authors properly reference that fact. The novelty of this work is mainly the analysis of the model in terms of bifurcation theory.

Thus, the work is of interest for applied mathematicians working in mathematical biology but is not accessible for physiologists or neuroscientists, and thus of little interest for a wider public. This could probably be improved by providing plots of membrane voltage vs time.

Unfortunately, not enough information is provided to reproduce the results shown.

Below is a detailed list of comments in order of appearance in the manuscript

Abstract : “... in determining a cold sensing neuron’s degree of response.”

How is the neuron's degree of response determined?

Line 41 “…, this model did not allow for the tracking of specific ion currents and thus investigation of the high versus low threshold question.”

The model in the manuscript does not track specific ion currents either. See below

Line 47 “1) to explicitly track differences in temperature response in relation to having different

ion channel densities”

Temperature responses should be shown in time domain also to make the work accessible for non-specialists

Line 52. Material and methods should include a reference to the software used.

Reproducible research should include the scripts used in generating the figures shown in Results.

Line 54: “The model is composed of a general Hodgkin-Huxley neuron taken from Byrne and

Roberts”

Quote also, or instead, the original paper by HH

Line 56:

The model only includes Hodgking-Huxley channels and TRPM8. But cold sensitive neurons have many more types of channels specifically voltage-dependent Ca channels and much experimental evidence is obtained my measuring intracellular calcium concentration changes.

The physiological relevance of this simplified model should be dicussed.

Minor point: in all formulas, symbols for sodium, and potassium should be Na and K, no “na” and “k”

Line 73: Vr should be -65mV, no 65mV

Lines 74-75: The value of the parameter Iinput is not specified

Formula (13) is incorrect, the same symbol T is used to represent temperature in Celsius in the numerator, and in Kelvin in the denominator, that is clearly wrong.

Line 86 “Note that the parameters of interest are gm8 , gk , and T, ...”,

However, gL and mainly gNa are also very important to determine the threshold in HH model, but have not been explored

Line 88: “The parameter Em8 will be discussed later with regards to its definition ...”

But it is never again mentioned in the manuscript, neither is its value specified.

Line 94 Fig 1., and all others should also show V(t) for each region if a wider audience is to be reached

Line 118 Fig. 2b Change black color in region 3 to another color (yellow, brown?) to avoid confusion with vertical black dotted lines. Same for Figs 4 and 6

Line 144 “keeping otherwise standard Hodgkin-Huxley parameter values, increasing g m8 shifts the neuron from high to a lower threshold (Fig 3, Fig 4)”

Relate this with the fact that TRPM8 has a reversal potential close to 0 mV

Line 146 “This shows that decreasing gk shifts the Hopf and SNP curves further right thus requiring a lower drop in temperature in order to turn on.”

Relate this with the fact that gk has an equilibrium potential at -77 mV

Line 152 “the width of the curves decreases as temperature decreases”

Relate this with the fact that the shape of am8 vs T, with a negative slope that increases as T decreases.

Line 163 “The goal here was to create a physiological model that would allow for investigation into the roles and properties of the involved ion channels and how they interact with changes in temperature.”

Yet only K and TRPM8 were explored, not Na nor Leak channels

Line 172 “The defining characteristic that separated high from low threshold was found to be the effectiveness of the TRPM8 channels. With a higher density of TRPM8 channels, the neuron can activate at higher temperature values.”

To be more specific show a graph of Threshold vs TRM8 density

Line 184 “By utilizing Hodgkin-Huxley as a basis for the model, both sodium and

potassium fluxes were able to be explicitly tracked.”

Yet no effect of gNa is shown

Lines 187-192 “Lowering the maximal conductance of potassium channels g k ,...”

The inverse effect of gK and g8 should also be discussed in relation to its reversal potentials

Reviewer #2: Numerical simulations of a sensory thermoreceptor are presented. The simulation comprised a single spatial compartment with Hodgkin-Huxley conductances and an additional TRPM8 conductance. Simulation of variations in temperature on H-H conductances alone, i.e. without TRPM8, revealed oscillatory behaviour occurring over a wide temperature range but only for very low values of the potassium conductance. The range of potassium conductance values and temperatures within which stable oscillatory behaviour is observed, enlarges with the addition of an explicitly temperature dependent TRPM8 conductance. The threshold temperature for the transition to stable oscillatory behaviour is shown to vary over a wide range commensurate with adjustment of the relative contributions of the potassium conductance (gK) and the TRPM8 conductance (gm8).

Using a framework distinct from the more common Huber-Braun formulation this dataset establishes that a simple combination of TRPM8 and standard HH conductances can replicate, prima facie, some features akin to firing behaviour for cold-sensitive neurons. A primary concern however is that the model, at least as presented, offers no justification that the stable oscillation result bears any resemblance to the firing patterns of cold receptors. This obscures the claim that transitions into stable oscillations actually represent, as the authors imply, an index of threshold. In addition, the implementation of simulation to investigate variations in temperature is not adequately described.

Each of these points are outlined in detail below.

Major points:

1. A clarification of the time course and amplitude profile of the oscillations in membrane potential, both stable and unstable, would be helpful to assess whether oscillations are physiologically meaningful. For example, are the oscillations sub-threshold oscillations in membrane potential, i.e. a few millivolts below AP firing, or simply action potential transitions driven say by a steady state inward current? The amplitude for stable oscillations indicated in Figure 2 suggest the latter. In either case, the frequency of the oscillation(s) is paramount to ascertain their physiological relevance. Perhaps some simple plots of membrane potential against time could resolve this.

2. The manuscript provides no clear definition of threshold. The reader must simply accept that a transition/bifurcation from a steady state membrane potential to an ill-defined oscillation (see point 1) represents something meaningful in terms of temperature coding. Threshold as it relates to the transition requires clarification and some degree of interpretation with regard to the biology that it is trying to emulate.

3. It is unclear which type of cold neuron is being simulated. Principally there are two forms of sensory neurons that encode decreases in temperature (see review by Vriens et al, Nat Rev Neurosci 2014). The canonical and most abundant form of cold-sensitive neuron exhibits ongoing activity at “normal” skin temperature and can encode both dynamic and static aspects of temperature stimuli over a wide range. The second broad grouping of cold receptor are those that respond below a certain temperature threshold but often provide little encoding of actual temperature and are often termed cold nociceptors.

If the simulation aims to replicate firing for the first class then it does not perform particularly well. By this I mean that the range of temperatures over which oscillatory behaviour manifests in the simulation does not reflect the temperature range of this type of cold receptor in mammals. The only version of the simulation getting anywhere near ongoing activity at physiological skin temperatures around 32°C is, ironically, the HH formulation without gm8 (Figure 1) or with very low gm8 (Figure 5).

4. How were variations in the temperature parameter actually implemented? This needs to be explicitly detailed in the manuscript. It appears that there are at least two possibilities here. For simulations, it is possible to simply start at any given temperature and run the simulation in time at this fixed starting temperature, then choose another starting temperature. This however would not reflect the biology. In physiological systems skin temperature is around 32°C and changes occur from this starting point. Since the simulation relies heavily on a concept of threshold, it would seem appropriate to evaluate changes in temperature simulated as they occur in nature, i.e. descending over time from a defined starting point.

Minor points:

1. The manuscript is not adequately referenced. Please ensure that a citation is provided for each explicit reference to previously published data. For example,

pp.6, l.169: “This coincides with lab findings highlighting the importance of TRPM8 channels within the class of cold thermosensors.“ citation to clarify which lab!

pp.6, l.171: „Previous work also highlights the variety of cold sensing neurons describing a range from high to low threshold neurons.” citation to indicate source of previous work!

2. In what software environment was the simulation implemented?

3. The code and resources should be made available via a public repository.

**********

6. PLOS authors have the option to publish the peer review history of their article (what does this mean?). If published, this will include your full peer review and any attached files.

If you choose “no”, your identity will remain anonymous but your review may still be made public.

**Do you want your identity to be public for this peer review?** For information about this choice, including consent withdrawal, please see our Privacy Policy.

Reviewer #1: No

Reviewer #2: No

[NOTE: If reviewer comments were submitted as an attachment file, they will be attached to this email and accessible via the submission site. Please log into your account, locate the manuscript record, and check for the action link "View Attachments". If this link does not appear, there are no attachment files to be viewed.]

While revising your submission, please upload your figure files to the Preflight Analysis and Conversion Engine (PACE) digital diagnostic tool, https://pacev2.apexcovantage.com/. PACE helps ensure that figures meet PLOS requirements. To use PACE, you must first register as a user. Registration is free. Then, login and navigate to the UPLOAD tab, where you will find detailed instructions on how to use the tool. If you encounter any issues or have any questions when using PACE, please email us at figures@plos.org. Please note that Supporting Information files do not need this step.

# Author comment: Author response to Decision Letter 0

**DOI:** 10.1371/journal.pone.0237347.r002

22 Jun 2020

Editor Comments:

1. Please ensure that your manuscript meets PLOS ONE's style requirements, including those for file naming. The PLOS ONE style templates can be found at https://journals.plos.org/plosone/s/file?id=wjVg/PLOSOne_formatting_sample_main_body.pdf and https://journals.plos.org/plosone/s/file?id=ba62/PLOSOne_formatting_sample_title_authors_affiliations.pdf

We have altered the title page to conform with the style requirements. We also have submitted Figures and manuscripts with the appropriate naming convention.

Reviewer 1 Comments

Abstract : “... in determining a cold sensing neuron’s degree of response.”

How is the neuron's degree of response determined?

Through bifurcation analysis we examine when and how the neuron activates and inactivates.

Line 41 “…, this model did not allow for the tracking of specific ion currents and thus investigation of the high versus low threshold question.”

The model in the manuscript does not track specific ion currents either. See below

We now have bifurcation analysis of the sodium and leak current interactions with temperature in figures 12-14. We show that without additional TRPM8 current, there is no temperature dependence at physiological (Hodgkin-Huxley) values for these current densities. With an added TRPM8 current we show that changing the sodium and leak currents can finely tune the temperature range in which the neuron would exhibit oscillatory behavior.

Line 47 “1) to explicitly track differences in temperature response in relation to having different

ion channel densities”

Temperature responses should be shown in time domain also to make the work accessible for non-specialists

Figures 4 and 5 now provide some membrane voltage plots against time to show the oscillations in response to temperature decrease. We have shown responses to both a simulated temperature ramp and fixed temperatures.

Line 52. Material and methods should include a reference to the software used.

Reproducible research should include the scripts used in generating the figures shown in Results.

Line 54: “The model is composed of a general Hodgkin-Huxley neuron taken from Byrne and

Roberts”

Quote also, or instead, the original paper by HH

Cited the original paper.

Line 56:

The model only includes Hodgkin-Huxley channels and TRPM8. But cold sensitive neurons have many more types of channels, specifically voltage-dependent Ca channels and much experimental evidence is obtained by measuring intracellular calcium concentration changes.

The physiological relevance of this simplified model should be discussed.

The purpose of this analysis is to provide insight for how temperature sensitivity can be regulated in a generic way. Most likely there are other ways that the same regulation can be accomplished, (i.e., other types of ion channels), but the same basic interplay of inward (TRPM8) and outward (g_k) currents is likely to be at play. 

Minor point: in all formulas, symbols for sodium, and potassium should be Na and K, no “na” and “k”

Fixed

Line 73: Vr should be -65mV, no 65mV

Fixed.

Lines 74-75: The value of the parameter Iinput is not specified

We’ve deleted I_input as we never apply an additional external input current to the system in our analysis.

Formula (13) is incorrect, the same symbol T is used to represent temperature in Celsius in the numerator, and in Kelvin in the denominator, that is clearly wrong.

Adjusted the formula so that all temperatures are in Celsius.

Line 86 “Note that the parameters of interest are gm8 , gk , and T, ...”,

However, gL and mainly gNa are also very important to determine the threshold in HH model, but have not been explored

We have added bifurcation analyses showing the relationship of gl and gNa with temperature.

Line 88: “The parameter Em8 will be discussed later with regards to its definition ...”

But it is never again mentioned in the manuscript, neither is its value specified.

We’ve cited and explained what the value of Em8 that we are using.

Line 94 Fig 1., and all others should also show V(t) for each region if a wider audience is to be reached

Figure 4 showcases a representation of V(t) in response to changing of temperature. Then the one parameter slices highlight the important features of when the neurons activate and inactive and the manner in which they do so.

Line 118 Fig. 2b Change black color in region 3 to another color (yellow, brown?) to avoid confusion with vertical black dotted lines. Same for Figs 4 and 6

We have noted in figure captions that the black dotted lines divide the regions.

Line 144 “keeping otherwise standard Hodgkin-Huxley parameter values, increasing g m8 shifts the neuron from high to a lower threshold (Fig 3, Fig 4)”

Relate this with the fact that TRPM8 has a reversal potential close to 0 mV

We have included some membrane potential plots that also track the TRPM8 current in Figure 4. These show for a fixed value of g_m8 how temperature changes affect the behavior of the neuron.

Line 146 “This shows that decreasing gk shifts the Hopf and SNP curves further right thus requiring a lower drop in temperature in order to turn on.”

Relate this with the fact that gk has an equilibrium potential at -77 mV

We now touch on the reversal potentials of the TRPM8 channels and potassium channels in establishing the threshold levels in the discussion. In particular we discuss how the offsetting effects these channels have are a result of their current type (inward, outward).

Line 152 “the width of the curves decreases as temperature decreases”

Relate this with the fact that the shape of am8 vs T, with a negative slope that increases as T decreases.

In figure 4 we emphasize how the TRPM8 current changes in response to temperature changes for fixed levels of g_m8.

Line 163 “The goal here was to create a physiological model that would allow for investigation into the roles and properties of the involved ion channels and how they interact with changes in temperature.” Yet only K and TRPM8 were explored, not Na nor Leak channels

We’ve now examined these currents and their influence on threshold level and general temperature dependence.

Line 172 “The defining characteristic that separated high from low threshold was found to be the effectiveness of the TRPM8 channels. With a higher density of TRPM8 channels, the neuron can activate at higher temperature values.”

To be more specific show a graph of Threshold vs TRM8 density

The bifurcation analysis of gm8 versus Temperature shows the threshold level of activation and inactivation for these cold sensitive neurons. 

Line 184 “By utilizing Hodgkin-Huxley as a basis for the model, both sodium and

potassium fluxes were able to be explicitly tracked.”

Yet no effect of gNa is shown

Lines 187-192 “Lowering the maximal conductance of potassium channels g k ,...”

The inverse effect of gK and g8 should also be discussed in relation to its reversal potentials

We emphasize that the inverse effect is also present in the bifurcation diagrams and discuss the reversal potentials as they relate to current type (inward, outward).

Reviewer 2 Comments

1. A clarification of the time course and amplitude profile of the oscillations in membrane potential, both stable and unstable, would be helpful to assess whether oscillations are physiologically meaningful. For example, are the oscillations sub-threshold oscillations in membrane potential, i.e. a few millivolts below AP firing, or simply action potential transitions driven say by a steady state inward current? The amplitude for stable oscillations indicated in Figure 2 suggest the latter. In either case, the frequency of the oscillation(s) is paramount to ascertain their physiological relevance. Perhaps some simple plots of membrane potential against time could resolve this.

We’re provided some plots of membrane potential against time. In particular we show the response of the neuron to a temperature ramp, showcasing what these oscillations look like and how the neuron jumps from steady state to oscillatory solutions. We also show what the neuron’s action potentials look like with a fixed g_m8 level and changing temperature levels. In addition we show how the changes in the action potentials and oscillatory behavior correspond to changes in TRPM8 current. 

2. The manuscript provides no clear definition of threshold. The reader must simply accept that a transition/bifurcation from a steady state membrane potential to an ill-defined oscillation (see point 1) represents something meaningful in terms of temperature coding. Threshold as it relates to the transition requires clarification and some degree of interpretation with regard to the biology that it is trying to emulate.

We have addressed this in the second paragraph in the introduction. We define threshold to be the temperature level at which the neuron activates. We are interested in exploring when the neurons turn on and off, and what influences this behavior. Furthermore we explore how well tuned this temperature response can be by exploring the width of the temperature responsive windows.

3. It is unclear which type of cold neuron is being simulated. Principally there are two forms of sensory neurons that encode decreases in temperature (see review by Vriens et al, Nat Rev Neurosci 2014). The canonical and most abundant form of cold-sensitive neuron exhibits ongoing activity at “normal” skin temperature and can encode both dynamic and static aspects of temperature stimuli over a wide range. The second broad grouping of cold receptors are those that respond below a certain temperature threshold but often provide little encoding of actual temperature and are often termed cold nociceptors.

If the simulation aims to replicate firing for the first class then it does not perform particularly well. By this I mean that the range of temperatures over which oscillatory behaviour manifests in the simulation does not reflect the temperature range of this type of cold receptor in mammals. The only version of the simulation getting anywhere near ongoing activity at physiological skin temperatures around 32°C is, ironically, the HH formulation without gm8 (Figure 1) or with very low gm8 (Figure 5).

The simulation focuses mainly on the second broad grouping of cold nociceptors. We aim to give a general description of how temperature sensitivity may be encoded. The analysis emphasizes the activation and inactivation temperatures for these neurons and shows that this model can explain a gradient of temperature windows for which these neurons will oscillate. 

4. How were variations in the temperature parameter actually implemented? This needs to be explicitly detailed in the manuscript. It appears that there are at least two possibilities here. For simulations, it is possible to simply start at any given temperature and run the simulation in time at this fixed starting temperature, then choose another starting temperature. This however would not reflect the biology. In physiological systems skin temperature is around 32°C and changes occur from this starting point. Since the simulation relies heavily on a concept of threshold, it would seem appropriate to evaluate changes in temperature simulated as they occur in nature, i.e. descending over time from a defined starting point.

In figure 4 we provide a simulated temperature ramp experiment that takes the neuron and exposes it to decreasing and then increasing levels of temperature. In figure 5 we expose the neuron to varying fixed temperature levels. For the bifurcation analysis we used XPPAUT, a numerical continuation method. This method continuously varies the parameters of interest (the different maximal conductances and temperature) and then examines the solution type (steady state, oscillatory). Thus for diagrams, moving through parameter space into region III the neuron transitions to firing action potentials.

Minor points:

1. The manuscript is not adequately referenced. Please ensure that a citation is provided for each explicit reference to previously published data. For example,

pp.6, l.169: “This coincides with lab findings highlighting the importance of TRPM8 channels within the class of cold thermosensors.“ citation to clarify which lab!

pp.6, l.171: „Previous work also highlights the variety of cold sensing neurons describing a range from high to low threshold neurons.” citation to indicate source of previous work!

We have provided more citations and cited statements that needed to be cited.

2. In what software environment was the simulation implemented?

The temperature ramp and fixed temperature simulations were done using MATLAB, the bifurcation analysis uses XPPAUT.

3. The code and resources should be made available via a public repository.

XPPAUT is interactive software for which no explicit code is generated.

# Peer review: Decision Letter 1

**DOI:** 10.1371/journal.pone.0237347.r003

**Authors:** David D McKemy

10 Jul 2020

PONE-D-20-10272R1

A mathematical model analyzing temperature threshold dependence in cold sensitive neurons

PLOS ONE

Dear Dr. McGahan,

Thank you for submitting your manuscript to PLOS ONE. After careful consideration, we feel that it has merit but does not fully meet PLOS ONE’s publication criteria as it currently stands. Therefore, we invite you to submit a revised version of the manuscript that addresses the points raised during the review process.

Specifically, please address Reviewer 1 remaining concerns a outlined in that review. 

Please submit your revised manuscript by Aug 24 2020 11:59PM. If you will need more time than this to complete your revisions, please reply to this message or contact the journal office at plosone@plos.org. When you're ready to submit your revision, log on to https://www.editorialmanager.com/pone/ and select the 'Submissions Needing Revision' folder to locate your manuscript file.

Please include the following items when submitting your revised manuscript:

- A rebuttal letter that responds to each point raised by the academic editor and reviewer(s). You should upload this letter as a separate file labeled 'Response to Reviewers'.
- A marked-up copy of your manuscript that highlights changes made to the original version. You should upload this as a separate file labeled 'Revised Manuscript with Track Changes'.
- An unmarked version of your revised paper without tracked changes. You should upload this as a separate file labeled 'Manuscript'.

If you would like to make changes to your financial disclosure, please include your updated statement in your cover letter. Guidelines for resubmitting your figure files are available below the reviewer comments at the end of this letter.

If applicable, we recommend that you deposit your laboratory protocols in protocols.io to enhance the reproducibility of your results. Protocols.io assigns your protocol its own identifier (DOI) so that it can be cited independently in the future. For instructions see: http://journals.plos.org/plosone/s/submission-guidelines#loc-laboratory-protocols

We look forward to receiving your revised manuscript.

Kind regards,

David D McKemy

Academic Editor

PLOS ONE

[Note: HTML markup is below. Please do not edit.]

Reviewers' comments:

Reviewer's Responses to Questions

**Comments to the Author**

1. If the authors have adequately addressed your comments raised in a previous round of review and you feel that this manuscript is now acceptable for publication, you may indicate that here to bypass the “Comments to the Author” section, enter your conflict of interest statement in the “Confidential to Editor” section, and submit your "Accept" recommendation.

Reviewer #1: (No Response)

Reviewer #2: All comments have been addressed

**********

2. Is the manuscript technically sound, and do the data support the conclusions?

The manuscript must describe a technically sound piece of scientific research with data that supports the conclusions. Experiments must have been conducted rigorously, with appropriate controls, replication, and sample sizes. The conclusions must be drawn appropriately based on the data presented. 

Reviewer #1: Yes

Reviewer #2: Yes

**********

3. Has the statistical analysis been performed appropriately and rigorously? 

Reviewer #1: Yes

Reviewer #2: N/A

**********

4. Have the authors made all data underlying the findings in their manuscript fully available?

The PLOS Data policy requires authors to make all data underlying the findings described in their manuscript fully available without restriction, with rare exception (please refer to the Data Availability Statement in the manuscript PDF file). The data should be provided as part of the manuscript or its supporting information, or deposited to a public repository. For example, in addition to summary statistics, the data points behind means, medians and variance measures should be available. If there are restrictions on publicly sharing data—e.g. participant privacy or use of data from a third party—those must be specified.

Reviewer #1: No

Reviewer #2: No

**********

5. Is the manuscript presented in an intelligible fashion and written in standard English?

PLOS ONE does not copyedit accepted manuscripts, so the language in submitted articles must be clear, correct, and unambiguous. Any typographical or grammatical errors should be corrected at revision, so please note any specific errors here.

Reviewer #1: Yes

Reviewer #2: Yes

**********

6. Review Comments to the Author

Please use the space provided to explain your answers to the questions above. You may also include additional comments for the author, including concerns about dual publication, research ethics, or publication ethics. (Please upload your review as an attachment if it exceeds 20,000 characters)

Reviewer #1: Please, insert reference to XPPAUT software webpage

I still consider that the authors should make the relevant software available without restrictions upon publication of the work

Although the authors state that "XPPAUT is interactive software for which no explicit code is generated", they should, at least, upload the ".ode" files used by XPPAUT into their GitHub repository where they have uploaded the MATLAB file, or to the ModelDB repository (https://senselab.med.yale.edu/modeldb/).

There are more than 100 papers that have uploaded their XPP models to ModelDB.

Reviewer #2: (No Response)

**********

7. PLOS authors have the option to publish the peer review history of their article (what does this mean?). If published, this will include your full peer review and any attached files.

If you choose “no”, your identity will remain anonymous but your review may still be made public.

**Do you want your identity to be public for this peer review?** For information about this choice, including consent withdrawal, please see our Privacy Policy.

Reviewer #1: No

Reviewer #2: No

[NOTE: If reviewer comments were submitted as an attachment file, they will be attached to this email and accessible via the submission site. Please log into your account, locate the manuscript record, and check for the action link "View Attachments". If this link does not appear, there are no attachment files.]

While revising your submission, please upload your figure files to the Preflight Analysis and Conversion Engine (PACE) digital diagnostic tool, https://pacev2.apexcovantage.com/. PACE helps ensure that figures meet PLOS requirements. To use PACE, you must first register as a user. Registration is free. Then, login and navigate to the UPLOAD tab, where you will find detailed instructions on how to use the tool. If you encounter any issues or have any questions when using PACE, please email PLOS at figures@plos.org. Please note that Supporting Information files do not need this step.

# Author comment: Author response to Decision Letter 1

**DOI:** 10.1371/journal.pone.0237347.r004

21 Jul 2020

Please, insert reference to XPPAUT software webpage

I still consider that the authors should make the relevant software available without restrictions upon publication of the work

Although the authors state that "XPPAUT is interactive software for which no explicit code is generated", they should, at least, upload the ".ode" files used by XPPAUT into their GitHub repository where they have uploaded the MATLAB file, or to the ModelDB repository (https://senselab.med.yale.edu/modeldb/).

There are more than 100 papers that have uploaded their XPP models to ModelDB.

We have inserted a reference to Bard Ermentrout website where XPPAUT can be downloaded. We have also uploaded our XPPAUT code to GitHub as requested.

# Editor report: Decision Letter 2

**DOI:** 10.1371/journal.pone.0237347.r005

**Authors:** David D McKemy

24 Jul 2020

A mathematical model analyzing temperature threshold dependence in cold sensitive neurons

PONE-D-20-10272R2

Dear Dr. McGahan,

We’re pleased to inform you that your manuscript has been judged scientifically suitable for publication and will be formally accepted for publication once it meets all outstanding technical requirements.

Within one week, you’ll receive an e-mail detailing the required amendments. When these have been addressed, you’ll receive a formal acceptance letter and your manuscript will be scheduled for publication.

An invoice for payment will follow shortly after the formal acceptance. To ensure an efficient process, please log into Editorial Manager at http://www.editorialmanager.com/pone/, click the 'Update My Information' link at the top of the page, and double check that your user information is up-to-date. If you have any billing related questions, please contact our Author Billing department directly at authorbilling@plos.org.

If your institution or institutions have a press office, please notify them about your upcoming paper to help maximize its impact. If they’ll be preparing press materials, please inform our press team as soon as possible -- no later than 48 hours after receiving the formal acceptance. Your manuscript will remain under strict press embargo until 2 pm Eastern Time on the date of publication. For more information, please contact onepress@plos.org.

Kind regards,

David D McKemy

Academic Editor

PLOS ONE

Additional Editor Comments (optional):

Reviewers' comments:
